# Hyperbranched Polymer Dendrimers Embedded in Electrospun Nanofibers for Safe and Sustainable Antibacterial Filtration Materials

**DOI:** 10.3390/polym18030374

**Published:** 2026-01-30

**Authors:** Matej Buzgo, Baturalp Yalcinkaya, Miroslav Doupník, Radmila Žižková, Viktorie Rockova, Kristyna Vrbova, Michaela Sobotkova, Alena Milcova, Anezka Vimrova, Michal Šíma, Pavel Rossner, Jamie Godfrey, Pedro Ferreira Costa, Amir Fahmi, Viraj Pratap Nirwan, Thomas Martinez, Eva Filová

**Affiliations:** 1 Respilon Membranes s.r.o., Nové Sady 988/2, Staré Brno, 602 00 Brno, Czech Republic; b.yalcinkaya@respilon.com (B.Y.);; 2 Institute of Experimental Medicine of the Czech Academy of Sciences, Vídeňská 1083, 142 20 Prague, Czech Republic; 3 Polymer Factory Sweden A.B., Teknikringen 48 1 TR, 114 28 Stockholm, Sweden; 4 Biofabics Lda, Rua Alfredo Allen 455, 4200-135 Porto, Portugal; 5 Faculty of Technology and Bionics, Rhine-Waal University of Applied Sciences, 47533 Kleve, Germany; 6 Leitat, C. de la Innovació, 2, 08225 Terrassa, Spain

**Keywords:** functional nanofibers, electrospinning, hyper-branched polymers, sustainable filters, antibacterial membranes

## Abstract

The global crisis concerning multidrug-resistant microorganisms necessitates the development of innovative antimicrobial strategies that avoid conventional antibiotics and overcome the toxicity and environmental persistence associated with traditional metal-based biocides. This work aims to develop safe and sustainable antibacterial filtration materials by integrating cationic hyperbranched polymer dendrimers (HBP) into electrospun nanofibers. Cationic HBPs were successfully embedded into recycled polyamide 6 nanofibers using industrial needleless electrospinning. Filtration efficiency, assessed against a 0.3 µm paraffin oil aerosol according to EN 149:2001, consistently exceeded 99.8%, meeting and surpassing the FFP3 classification threshold while maintaining low air resistance. The HBP-functionalized nanofibers exhibited pronounced contact-active antibacterial activity against *Staphylococcus aureus* and *Escherichia coli*. Quantitative plate count assays confirmed viability reductions of up to 74.1% after 2 h of co-incubation. Crucially, the absence of inhibition zones in agar diffusion tests confirmed that the active polymer was stably embedded within the nanofiber matrix and did not leach. Comprehensive toxicological tests, including cell line and 3D human skin and airway tissue models, confirmed the material’s safety for both dermal and respiratory contact. This study presents a scalable, metal-free, and environmentally responsible next-generation filtration system that combines high mechanical efficiency with active antimicrobial functionality.

## 1. Introduction

Microorganisms resistant to multiple drugs are a global health issue in the 21st century. Antibiotic-resistant bacterial infections likely claimed 1.27 million lives in 2019 [1]. If current trends continue, antimicrobial resistance (AMR) could claim the lives of 10 million people annually by 2050 [2]. Antibiotic misuse, overuse, and slow drug development are contributing to the problem [3]. Antibiotic resistance has outpaced the development of antibiotics, leaving a gap in treatment options. Due to antibiotic shortages, common infections are becoming increasingly difficult to treat, and everyday medical procedures are becoming riskier [4]. Thus, non-antibiotic antimicrobial strategies are urgently needed.

Silver, copper, and zinc-based antimicrobials are popular alternatives to antibiotics [5]. These broad-spectrum biocidal metals are utilized in medical devices, coatings, textiles, and filtration systems to combat microbial growth. Silver nanoparticles (AgNPs) are used in many consumer and healthcare products due to their antimicrobial properties [6]. However, metal-based strategies pose safety and sustainability issues [7,8]. Heavy metal ions are non-selective toxins. Even essential metals like Cu^2+^ and Zn^2+^ can damage human cells at high concentrations, disrupting cellular equilibrium [9]. The environmental impact of metal antimicrobials is growing. Consumer products can leach metal nanoparticles, posing a threat to ecosystems [10]. Moreover, research shows that silver nanoparticles (AgNPs) can harm non-target organisms [11]. Daphnia magna, a freshwater crustacean, shows acute toxicity at AgNP concentrations of 0.03 µg/mL [12]. There is also evidence that microbial populations can develop heavy metal tolerance or co-resistance, reducing their long-term effectiveness [13]. Researchers and regulatory bodies are increasingly advocating for non-toxic [14], effective biocides due to these concerns [12,15].

The “Safe and Sustainable by Design” (SSbD) framework centers on this idea. SSbD, or “sustainable by design,” is a growing concept in EU policy [16]. The aim is to engineer new materials and chemicals from the start to reduce human and environmental harm. A Safe-by-Design (SbD) approach requires developing antimicrobial solutions that perform as needed without toxicity or persistence issues [17]. Hyperbranched dendritic polymers, which follow SSbD principles [18], are promising antimicrobials [19]. Dendritic macromolecules are globular and highly branched and have many functional end groups. Dendritic polymers can have cationic or amphiphilic surfaces by selecting chemical processes [20,21]. These properties resemble natural antimicrobial peptides. Unlike enzymatic inhibitors, these macromolecular antimicrobials eliminate bacteria by disrupting membranes [22].

Dendritic and hyperbranched polyesters have broad-spectrum antibacterial and biocompatible properties, according to Malkoch and colleagues [23]. Fan et al. synthesized cationic bis-MPA (2,2-bis(methylol)propionic acid) dendritic-linear polymers that killed both Gram-positive and Gram-negative bacteria, including drug-resistant *Staphylococcus aureus* strains, in vitro, while causing minimal cytotoxicity to keratinocytes. Dendritic polymers feature dense cationic groups that bind to and disrupt microbial membranes, thereby bypassing the limitations of small-molecule antibiotics and achieving high killing efficacy [24,25]. Even after repeated bacterial exposures, they did not show resistance. These macromolecules can also be biodegradable, breaking down into harmless oligomers or monomers in the body. Biodegradability prevents them from accumulating in living things or the environment, ensuring sustainability [26]. Dendritic polymers are promising “safe-by-design” antibacterial agents due to their strong antimicrobial effects, low toxicity, and low environmental impact [27].

Electrospinning can produce ultrafine polymer fibers from a few tens of nanometers to a few microns [28]. The nanofibrous nonwoven membranes’ high surface-area-to-volume ratios and porosity make them exceptional filtration membranes [29]. Nanofiber mats capture nanoscale aerosol particles (even those smaller than the pore size) through inertial impaction, interception, Brownian diffusion, and electrostatic attraction, unlike conventional microfiber filters [30]. For particles ranging from 100 to 300 nm, including viruses such as SARS-CoV-2 (~160 nm), diffusion and interception are more effective than air streamlines for capture, as these tiny particles move randomly and collide with fibers [31]. Electrospun nanofibers form a fine web, increasing particle collisions. Therefore, nanofiber filters are effective at removing virus-sized particles [32,33]. Nanofiber membranes have high filtration efficiency and low pressure drop due to their highly interconnected pores, allowing air to flow while providing protection. Research indicates that sub-micron polyamide nanofiber layers can remove over 95% of 0.1–0.3 µm aerosols [34] at air resistances suitable for respiratory masks [35].

A single-needle emitter limits the throughput of conventional electrospinning [36]. Multineedle systems require precise alignment to maintain a uniform electric field, and the large number of needle emitters can clog [37]. However, needleless electrospinning systems utilize open liquid surfaces or multiple tips to generate numerous parallel jets, thereby increasing fiber output. Self-organization of fiber jets enables the system to automatically adjust their positions, promoting homogeneous distribution even when the properties of the polymeric solution vary, without requiring hardware modifications [38,39]. Thus, this method allows continuous roll-to-roll production of uniform nanofiber mats. Various spinneret designs can produce meter-wide nanofiber membranes in a single run, and industry-grade needleless electrospinning machines can produce hundreds of square meters of nanofiber web per hour [40]. Thousands of layers of facemasks and air filter cartridges are produced daily with consistent quality using the electrospinning technique [41]. Its scalability and consistency are distinct advantages over melt-blown microfibers. While melt-blowing is effective for producing filter media, such as N95 mask layers, it produces coarse fibers (1–10 µm in diameter) with larger pore sizes and requires post-charging for high fine-particle capture [31]. However, electrospun nanofibers naturally create smaller pores and can filter efficiently without charging [35].

Nanofiber-based filters are more stable and reusable than electret filters [42]. The fibers’ electrostatic charge helps melt-blown polypropylene filters capture particles in respirators. However, moisture, oils, or specific decontamination methods can reduce these charges, significantly reducing filtering [35,43]. Due to electrostatic charge loss, disinfecting a N95 melt-blown layer with ethanol reduced aerosol filtration efficiency from 95% to 64% [35]. Nanofiber filters use mechanical interception and diffusion rather than permanent electrostatic charges. Nanofiber filters have lower airflow resistance than comparable melt-blown filters. This is due to nanoscale slip-flow effects and the nanoweb’s open pore structure [43]. Thus, face masks are more breathable and comfortable without sacrificing protection. Current nanofiber membranes lack antimicrobial properties, limiting their reuse and extended use [44].

The present work explores the use of hyperbranched polymer dendrimers embedded in electrospun nanofibers as a pathway toward the development of safe and sustainable antibacterial filtration materials. Using dendritic antimicrobial polymers and nanofibrous membranes to create a self-disinfecting filter increases filtration efficiency and biocidal activity. In this model, the dendritic polymer’s antibacterial surface neutralizes airborne bacteria and viruses captured by the filter, preventing pathogen accumulation. Thus, this self-cleaning mechanism reduces pathogen release and secondary contamination during filter handling. By electrospinning dendritic macromolecules into nanofiber membranes, we can create high-performance, antibacterial air filters that meet SSbD standards. These filters would not contain leachable metallic particles or hazardous additives, and the active polymer can be engineered to remain fixed within the fibers or degrade into non-toxic byproducts, ensuring environmental safety.

## 2. Materials and Methods

### 2.1. Synthesis of Hyperbranched Dendritic Polymers

A range of dendritic polymers was produced to function as antimicrobial additives for the electrospun fibers. HBPs are made by growing polymeric layers, or generations, in which each generation yields exponentially more end groups that can be equipped with chemical groups to impart specific functions. The polymers used were synthesised as previously reported [23]. Briefly, hydroxyl-terminated generation 4 or 5 HBPs with PEG10k core were produced by melting the PEG10k (Mn: 10,000, Sigma-Aldrich, Merck, Darmstadt, Germany) core at 130 °C before the addition of 2 equivalents of 2,2-Bis(hydroxymethyl)propionic acid (Mw: 134.13, Purity: 97.5–102.5% Sigma Aldrich, Merck, Darmstadt, Germany) (bis-MPA) and p-Toluenesulfonic acid, (Mw: 190.22, Purity ≥ 98.5% Sigma-Aldrich, Merck, Darmstadt, Germany) (pTSA) (5 wt.% to bis-MPA) and subjecting the reaction mixture to vacuum for 1 h. The bis-MPA equivalents corresponding to the next generation (i.e., 4, 8, 16, 32 equiv.) and pTSA (5 wt.% to bis-MPA) were added until the desired generation was reached. Subsequently, a vacuum was applied, and stirring was continued at 130 °C for 16–18 h. The hydroxyl-terminated HBPs were converted to boc-protected beta-alanine esters by treatment with an excess of CDI-activated boc-β-ala-OH (Purity: ≥98.0%, Mw: 189.21, MCE, Monmouth Junction, NJ, USA). Deprotection with trifluoroacetic acid (Purity: ≥99.0%, Sigma-Aldrich, Merck, Darmstadt, Germany) followed by precipitation into diethyl ether and drying to a constant mass afforded the amine-terminated HBPs.

### 2.2. Solution Preparation for Electrospinning of HBPs

Two distinct steps have been employed to prepare solutions of HBPs and polyamide 6 (PA6). Aquafil S.p.A supplied Recycled PA6 (Econyl^®^ Recycled 27). The manufacturer does not publicly publish a “typical molecular weight (Mn/Mw)” for ECO-NYL-recycled PA6 (Arco, Italy). First, 20 g of PA6 was dissolved in 100 mL of acetic acid (AA): formic acid (FA): chloroform (CH) (Purity AA: 99.7%, FA: 98%, CH: 99% VWR International s.r.o., Prague, Czech Republic) (2:2:1 *v*/*v*) by heating to 50 °C and stirring overnight. HPB powder was dissolved in the same solution (30 wt.% stock) and mixed with the PA6 solution to obtain spinning solutions with the desired composition. The solution was mixed using a magnetic stirrer (VELP ARE F20500162, VELP Scientifica Srl, Valeta, MB, Italy) for 30 min at room temperature until completely homogenized.

### 2.3. Electrospinning of Pristine PA6 and Hybrid PA6/HBP Nanofibers

**Figure 1:**
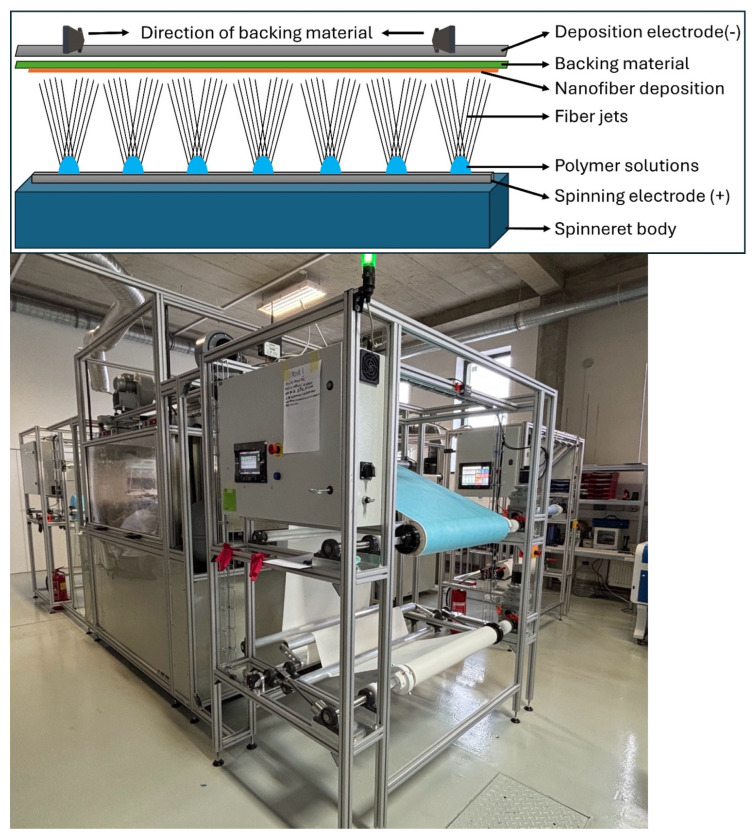
Needleless electrospinning spinneret body and spinning process. Adapted from [38], Polymers, 2025.

Polyamide 6 pristine and hybrid nanofibers were electrospun using needleless electrospinning (Figure 1). The electrospinning system utilised in this study is an industrial-scale machine (NEUNEX, RESPILON, Prague, Czech Republic) with a width of 80 cm and a length of 2 m, as previously described [15]. Before nanofiber production, it is essential to maintain the electrospinning chamber at specific humidity and temperature levels. These environmental conditions are regulated by an integrated air-drying system (RH 20–25%, temperature 22–24 °C). The industrial-scale needleless electrospinning setup operates with a roll-to-roll nanofiber deposition system (Figure 1).

All samples were produced using a linear electrode, operating on the principle that the polymeric solution is deposited on the surface of a 2 mm-thick metallic blade measuring 80 cm in length. The solution is dispersed using a moving element, enabling constant feeding of fresh polymeric solution to the electrospinning zone. The feeding head velocity was set to 100 mm/min, and the polymer pump pressure was set to 75 mbar. The electrode was connected to a high-voltage source at 70 kV. The counter electrode was connected to a negative high-voltage source at −29 kV. A 45 gsm polypropylene/meltblown nonwoven substrate is loaded into the unwinder unit and fed into the spinning chamber, where nanofibers are deposited onto the backing material. The rewinder unit then collects the coated nonwoven.

### 2.4. Characterisation of Nanofibers

The morphology and average diameter of the produced natural, synthetic, and hybrid nanofibers were analysed using scanning electron microscopy (SEM; Phenom ProX, Thermo Fisher Scientific, Waltham, MA, USA). To prepare the samples, nanofiber specimens with their nonwoven substrate were cut into appropriate dimensions (0.75 cm^2^) to ensure compatibility with the SEM sample holder. Since nanofibers on a nonwoven substrate are typically non-conductive, a conductive coating is necessary to minimise charging effects and enhance imaging quality. Therefore, a 5 nm gold layer was deposited onto the samples using a LUXOR-AU sputter coater (Aptco Technologies, Luxor Tech, Mechelen, Belgium), ensuring optimal conductivity for high-resolution SEM imaging. The mean fiber diameter, pore size, and porosity were calculated using ImageJ Software 1.54r.

The bulk polymer, HBPs, and the functionalized nanofibers were analyzed using a Shimadzu IRXross FTIR spectrometer (Shimadzu Corporation, Kyoto, Japan) to measure ATR-FTIR absorbance across 4000–500 cm^−1^ at a resolution of 2 cm^−1^.

### 2.5. Microbiological Testing

#### 2.5.1. Microbial Testing in Co-Incubation Experiment

For the plate count assay, nanofiber samples were co-incubated with CCM 2022 bacterial cells in 1 mL of phosphate buffer for 2 h at room temperature. The initial bacterial concentration was identical to that used in the diffusion assay to allow direct comparison between the two methods. After the co-incubation period, the bacterial suspension was immediately subjected to serial dilution and plated on agar using the standard plate count method to determine the number of viable cells (CFU/mL). Importantly, CFU enumeration was performed exclusively on the surrounding liquid phase following co-incubation. No additional detachment, washing, or sonication steps were applied to recover bacteria adhered to the nanofiber surface. Therefore, the measured reduction in bacterial counts reflects changes in bacterial viability within the suspension after direct contact with the nanofiber material, rather than a combined contribution from surface-adhered bacteria.

#### 2.5.2. Determination of Minimum Inhibitory Concentration (MIC) and Minimum Bactericidal Concentration (MBC)

Minimum Inhibitory Concentration (MIC) and Minimum Bactericidal Concentration (MBC) testing define a test material’s potency in terms of the concentration at which it will inhibit growth of (MIC) or completely kill (MBC) challenge microorganisms during an 18- to 20-h period of incubated exposure (35 ± 2 °C). MICs and MBCs of the materials listed in Table 1 were determined against *Staphylococcus aureus* (*S. aureus*) and *Escherichia coli* (*E. coli*). *E. coli* and *S. aureus* are commonly used as model organisms to determine MIC and MBC. *E. coli* is a classic Gram-negative bacterium, characterised by a thin peptidoglycan layer surrounded by an outer lipopolysaccharide membrane [45]. This structure makes it more resistant to certain compounds due to lower permeability. *S. aureus* is a Gram-positive bacterium with a thick peptidoglycan cell wall but no outer membrane, making it generally more susceptible to agents that target cell wall synthesis (e.g., beta-lactams). These differences make them good candidates for assessing a compound’s broad-spectrum potential or specificity against different bacterial architectures. Those bacteria frequently infect the human body and are responsible for various symptoms, including urinary tract infections, gastrointestinal issues, sepsis (in the case of *E. coli*), skin and soft tissue infections, pneumonia, and bloodstream infections (in the case of *S. aureus*). The product is serially diluted in a growth medium suitable for each of the challenge microorganisms. In the experiments reported in this study, the concentration usually used for polymer dissolution in electrospinning was chosen and diluted as follows: 0.8% initial concentration, 1:2 dilution to 0.4%, 1:10 dilution to 0.08%, and 1:100 dilution to 0.008%.

#### 2.5.3. Antibacterial Activity Evaluation by Adaptation of ISO20645:2005 Textile Fabrics

A plate disc diffusion test, adapted from ISO 20645 [46] “Textile fabrics determination of antibacterial activity, agar diffusion plate test”, was carried out on samples MB47.2 and MB47.4 (Table 1) to determine their bacterial growth inhibition on agar plates. Two strains of *E. coli* and *S. aureus* were used in all the antibacterial assays. The method consists of placing the sample in contact with nutrient agar plates containing bacterial cells. This qualitative method evaluates bacterial activity by halo formation (the absence of bacterial growth immediately around the edges of the samples) and by growth inhibition within the sample. All the samples used in antibacterial tests were sterilised before incubation with bacterial medium (agar plates and inoculated medium) using UVC light. This sterilisation method was chosen to avoid altering the nanofibers due to liquid or temperature. After 24 h of contact time, the inhibition zone, if present, is measured. The samples are removed from the agar, and the contact zones below the test specimens are examined under a 20× microscope. Table 1 below presents the classification of the biocide effect based on the growth of microorganisms around and above the sample.

### 2.6. Biocompatibility and Cytotoxicity Evaluation

#### 2.6.1. Cell Seeding

All cells were commercially available; 3T3-A21 mouse fibroblasts were supplied from Sigma-Aldrich, HaCaT human keratinocytes, and Melan-a mouse melanocyte cells from Welcome Trust Functional Genomics Cell Bank at St. George’s, University of London. BEAS-2B human bronchial epithelial cells were obtained from American Tissue Culture Collection (ATCC; CRL-9609TM, ATCC^®^, Manassas, VA, USA).

Cells were seeded 24 h before the addition of the extracts. Each cell line was seeded at a different concentration to ensure subconfluency on the day of the extract addition. The optimal seeding concentration for each cell type was optimized in advance. Mouse 3T3 fibroblasts were seeded at a concentration of 5000 cells/well of a 96-well plate, human HaCaT keratinocytes were seeded at a concentration of 8000 cells/well of a 96-well plate, mouse Melan-a melanocytes were seeded at a concentration of 12,000 cells/well of a 96-well plate, and human BEAS-2B bronchial epithelial cells were seeded at a concentration of 18,000 cells/well of a 96-well plate for 24-h experiment and 10,000 cells/well of a 96-well plate for 72-h experiment. All cell types were seeded at 200 µL, except BEAS-2B, which was seeded at 100 µL to improve cell adhesion.

3T3-A21 fibroblasts and HaCaT keratinocytes were maintained in Dulbecco’s Modified Eagle’s Medium (DMEM; cat. No. D6429, Sigma-Aldrich, St. Louis, MO, USA) supplemented with 10% fetal bovine serum (FBS; cat. No. 10270106, Gibco, Grand Island, NY, USA) and a penicillin–streptomycin solution providing 100 U/mL penicillin and 100 µg/mL streptomycin (cat. No. P4333, Gibco Grand Island, NY, USA). Melan-a melanocytes were cultured in RPMI-1640 medium (cat. No. R8758, Sigma-Aldrich, St. Luis, MO, USA) containing 10% FBS (cat. No. 10270106, Gibco, Grand Island, NY, USA), 100 U/mL penicillin and 100 µg/mL streptomycin (cat. No. P4333, Gibco, Grand Island, NY, USA), 2 mM L-glutamine (cat. No. 25030-024, Gibco), and phorbol 12-myristate 13-acetate (PMA; cat. No. P8139, Sigma-Aldrich, St. Luis, MO, USA). BEAS-2B bronchial epithelial cells were cultured using the BEGM™ BulletKit system (LONZA Bioscience, Basel, Switzerland, CC-3171 and CC-4175), consisting of BEBM™ Bronchial Epithelial Cell Growth Basal Medium (CC-3171) supplemented with the BEGM™ SingleQuots™ growth factors and additives (CC-4175) according to the manufacturer’s instructions. Cells were cultured in a 37 °C, high humidity, and 10% or 5% CO_2_ atmosphere in the case of skin cells or BEAS-2B cells, respectively.

Extracts from the materials were prepared 24 h before addition to cells in the appropriate cell culture medium, under conditions identical to those of cell culture. Materials were cut into squares measuring 8.5 cm^2^ and leached in 6.25 mL of culture medium in glass bottles with a partially unscrewed lid to ensure gas exchange. Culture medium incubated in the same conditions served as a negative control. After 24 h, 200 µL of the extract was added to the cells. The cells were incubated for the specified time, as indicated for the analysis: 24 and 72 h for metabolic activity measurement and Live/Dead assays, 1.5 h for the oxidative stress measurement, 24 h for the comet assay, 24 and 72 h for micronucleus assay, 24 and 48 h for analyses on EpiDerm FT™, and 24 h for analyses on MucilAir™.

#### 2.6.2. Metabolic Activity

Cytotoxicity of the extracts was analysed by measuring metabolic activity using the MTS assay (CellTiter 96^®^ Aqueous One Solution Cell Proliferation Assay; cat. No. G1111, Promega, Madison, WI, USA). At the desired time interval, 100 μL of fresh culture medium containing 20 µL of MTS solution was added to the cells, which were incubated at 37 °C for 2 h. Mitochondrial enzymes metabolised the MTS substrate to the violet formazan, which absorbs light at 490 nm. 100 µL of the metabolised solution was read in clean microplates using a microplate reader (Infinite M200 PRO, Tecan, Grödig, Austria, A-5082) at 490 nm. 6 replicates were used, with one well without cells serving as a background for the leachate.

#### 2.6.3. Live/Dead (L/D) Assay

Cell viability was assessed using fluorescent live/dead staining (L/D). 2,7′-Bis(2-carboxyethyl)-5(6)-carboxyfluorescein acetoxymethyl ester (BCECF-AM; cat. no. B8806, Sigma-Aldrich, St. Luis, MO, USA) probe was added to the cells at a concentration of 10 µM in culture medium without any supplements to stain live cells. The cells were incubated for 30 min at 37 °C in the dark. BCECF-AM is hydrolysed inside live cells by cytosolic esterase to BCECF, which emits light in the green part of the spectrum (λex/λem = 503/528 nm). Propidium iodide (PI; cat. no. P4864, Sigma-Aldrich, St. Luis, MO, USA) was added to the cells at 5 μg/mL in culture medium without supplements to stain dead cells. The cells were incubated for 10 min at 37 °C in the dark. PI does not penetrate the membrane of living cells, so it stains only the DNA of dead cells. Subsequently, samples were rinsed with culture medium and visualised using an Olympus IX70 fluorescence microscope with an Olympus DP80 camera and a 10× objective. Three parallel wells per group were seeded for the L/D staining.

#### 2.6.4. Oxidative Stress Measurement

H_2_O_2_ is a reactive oxygen species (ROS) that is created during oxidative stress. Production of H_2_O_2_ in extracts was analysed using ROS-Glo™ H_2_O_2_ Assay (Promega, Cat. No. G8820, Madison, WJ, USA). 80 µL of the extract, the control medium (negative control), or the medium containing 750 µM H_2_O_2_ (positive control) was added to the cells. Then, 20 µL of H_2_O_2_ substrate solution (prepared from 20 µL of H_2_O_2_ substrate dilution buffer + 0.25 µL of H_2_O_2_ substrate) was added, and the cells were incubated for 1.5 h at 37 °C. After incubation, 100 µL of ROS-Glo™ Detection Solution (prepared from 100 µL of Luciferin Detection Reagent, 1 µL of D-Cysteine, and 1 µL of Signal Enhancer Solution) was added. The cells were incubated for 20 min at room temperature. A microplate reader recorded the luminescence with no attenuation, a 500 ms integration time, and a 0 ms settle time.

#### 2.6.5. Micronucleus Assay

The micronucleus assay was applied as a sensitive method for in vitro detection of cytogenetic cell damage. DNA damage (losses or breaks of chromosomes) is manifested as micronuclei. These structures are visible in the cell cytoplasm during interphase, with staining characteristics similar to those of the main cell nucleus. They contain either whole chromosomes or chromosomal fragments. After the 24 h and 72 h exposures, the cells were washed with 2 mL of PBS (16 wells harvested together), then 320 µL of trypsin was added, and incubation was performed at 37 °C for about 4 min. Then the cells were resuspended in 2 mL of PBS and centrifuged at 20 °C for 5 min, 200 g. The supernatant was aspirated, the cell pellet resuspended, and 5 mL of 37 °C hypotonic solution (0.55% KCl in H_2_O) was added. Samples were incubated for 5 min at RT and then centrifuged for 5 min at 200× *g*. The supernatant was aspirated, the cells were resuspended and then fixed twice in fixative solution (methanol: acetic acid, 3:1). 3 mL of 4 °C fixative solution was added, and the cells were centrifuged for 5 min at 200× *g*. In the next step, 3 mL of 4 °C methanol was added while vortexing, and the mixture was centrifuged for 5 min at 200× *g*. The supernatant was aspirated to a small volume (0.5 mL). The samples were dropped onto slides, which were dried at RT, and stained with 5% Giemsa solution for 3–4 min. Then, the slides were rinsed thoroughly under running water and allowed to dry. The frequency of micronuclei in mononucleated cells was analysed at ×1000 magnification using an Olympus BX41 microscope (Tokyo, Japan) to assess staining efficiency and cell density. Two thousand mononucleated cells were scored in each sample. The results were expressed as a % of aberrant cells in 1000 mononucleated cells.

#### 2.6.6. Comet Assay with BEAS-2B Cell

DNA damage was analysed using an alkaline version of the enzyme-modified comet assay. The principle of the assay is that, under an electric field, fragmented DNA migrates out of the nucleoid body and forms a DNA stain in the agarose gel, also known as the “comet tail”. After the 24 h incubation, the cells were washed with warm PBS at 37 °C, trypsinised for 3–5 min, and 4 °C cold PBS was added to the cell suspension. Samples were centrifuged (200× *g*, 5 min, 4 °C), rewashed in cold PBS, centrifuged, and then frozen in freezing medium (BEGM:FBS:DMSO—5:4:1) at a concentration of 900,000 cells/mL. Immediately before application on slides, cells were quickly thawed in a 37 °C water bath. Two slides per sample were prepared—each with two gels. The slides were submerged in a lysing solution (2.5 M NaCl, 100 mM EDTA, 10 mM Tris, 0.16 M dimethyl sulfoxide, 0.016 mM Triton X-100; all Sigma-Aldrich) at pH 10 for 1 h, then washed with phosphate-buffered saline (3× for 5 min). After that, one slide (i.e., two gels) per sample was treated with formamidopyrimidine-glycosylase (FPG, New England Biolabs, Ipswich, MA, USA). Each gel was incubated with 50 µL of FPG (final concentration, 2.5 µg/mL; Sigma-Aldrich) for 30 min at 37 °C. In parallel, the remaining two gels per sample were treated with the same volume of buffer used for enzyme dilution (0.1 M KCl, 4 mM EDTA, 2.5 mM HEPES, 2% bovine serum albumin; all Sigma-Aldrich). Subsequently, the slides were equilibrated for 40 min in alkaline buffer (0.3 M NaOH, 1 mM EDTA, pH 13) to allow the DNA to unwind. Electrophoresis was performed in fresh alkaline buffer (30 min, 1 V/cm, 300 mA) at 4 °C. Finally, the slides were neutralised in 0.4 M Tris (pH 7.5), washed with distilled water (2 × 5 min), fixed in ethanol (15 min 70%; 15 min 99%), and dried at room temperature. The last step was staining with SYBR™ Safe DNA Gel Stain (Invitrogen™, Waltham, MA, USA) for 30 min. After drying (24 h, RT, dark), the slides were stored at 4 °C. Microscopic images were captured with a CCD-13008 camera (VDS, Vosskuhler, Osnabrück, Germany) attached to a BX51 fluorescence microscope (Olympus, Tokyo, Japan). The extent of DNA migration was quantified using Lucia Comet Assay 7.00 software (Laboratory Imaging, Prague, Czech Republic), and the results were expressed as the percentage of DNA in the tail (Tail DNA%). Both total DNA damage (with enzyme) and DNA strand breaks (without enzyme) were measured in 100 randomly selected cells per sample. Each sample was characterised by the median value of total DNA damage and the median value of DNA strand breaks. Oxidative stress was quantified as the difference between these medians.

### 2.7. Biocompatibility—Human 3D Tissue Models

#### 2.7.1. Evaluation of Reconstructed Human Upper Airway Model (MucilAir™)

Advanced toxicological testing was performed using the MucilAir™-Pool model (Epithelix Sàrl, Geneva, Switzerland), a well-differentiated 3D human nasal epithelium reconstructed from primary cells of 14 healthy donors and maintained at the air–liquid interface (ALI) in MucilAir™ culture medium at 37 °C, 5% CO_2_, and >90% relative humidity. Before exposure, cultures were stabilised for one week, with medium changes every 2–3 days and apical washes to remove mucus. The inserts were placed into a custom-built exposure system [47,48] comprising a humidified, temperature-controlled toxicological incubator (37 °C, 5% CO_2_, >90% RH) and exposure chambers connected to a flow of synthetic air (20% O_2_, 80% N_2_) passing through filter holders containing nanomaterials. Control samples were exposed to clean synthetic air under identical conditions. The exposure regimen consisted of a 2-h exposure, 2-h rest, and a second 2-h exposure. Transepithelial electrical resistance (TEER) was measured 24 h after exposure, followed by collection of basal medium for LDH assay and harvesting of cells for gene expression analysis and comet assay.

#### 2.7.2. Evaluation on Reconstructed Human Skin Model (EpiDerm FT™)

The EpiDermFT™ model (MatTek Corporation, Ashland, MA, USA) is a reconstructed full-thickness human skin equivalent composed of normal human epidermal keratinocytes and dermal fibroblasts, co-cultured to form a multilayered structure that closely mimics native human skin. The tissue inserts were handled according to the manufacturer’s protocol. Residual agarose was removed, and inserts were transferred into 6-well plates containing 2.5 mL of pre-warmed EFT-400-ASY Assay Medium (MatTek). The tissues were equilibrated for 18 h at 37 °C, under air–liquid interface (ALI) conditions with 5% CO_2_. Leachates of test materials were prepared by incubating 18.6 cm^2^ of each sample in 13.68 mL of assay medium for 24 h at 37 °C. The resulting leachates were applied to the apical surface of the EpiDermFT™ tissues (200 µL per insert). Pure assay medium served as a negative control, while 1% Triton X-100 solution applied to both apical and basal compartments 2 h before the assay served as a positive cytotoxicity control. Tissue viability and barrier integrity were analysed at 24 and 48 h after exposure using TEER and LDH assays as described below and cells were harvested for gene expression analysis, with four parallel tissue replicates per condition.

#### 2.7.3. Transepithelial Electrical Resistance (TEER)

The barrier integrity of the tissue models was quantitatively evaluated using transepithelial electrical resistance (TEER) measurements performed with an EVOM2 epithelial volt-ohm meter equipped with STX2 chopstick electrodes (World Precision Instruments, Sarasota, FL, USA). Measurements were conducted under sterile conditions at 37 °C to ensure cell viability. Before measurement, 200 µL of prewarmed MucilAir™ culture medium was added to the apical surface of each insert. For EpiDerm FT™, tissues were washed with PBS, and measurements were performed in PBS. TEER values (Ω·cm^2^) were calculated according to the formula:

TEER = (R_sample − R_blank) × A,

where R_sample is the measured resistance of the tissue, R_blank is the resistance of the insert without cells (100 Ω), and A is the surface area of the tissue model.

#### 2.7.4. Lactate Dehydrogenase (LDH)

Lactate dehydrogenase activity (LDH, an enzyme released during cell damage) was analysed as a marker of cytotoxicity using the Cytotoxicity Detection Kit (cat. no. 11644793001; Roche, Basel, Switzerland). Extracts or control culture medium incubated with cells for the desired time were mixed with the LDH detection kit at a 1:1 ratio and incubated for 30 min at 37 °C. The LDH detection kit is a mixture of catalyst and staining solution in a 1:45 ratio. Absorbance was measured at 490 nm.

#### 2.7.5. Comet Assay with MucilAir™

24 h after exposure, one insert per sample or control was harvested according to the following procedure. Inserts were placed into other wells, washed with warm PBS at 37 °C, and trypsinised for 3–5 min. Suspension from the apical side was preserved. The apical side was rewashed with cold PBS at 4 °C, and the suspensions were pooled. Consequently, samples were centrifuged (200× *g*, 5 min, 4 °C), rewashed in cold PBS, centrifuged, and frozen in freezing medium (BEGM:FBS:DMSO—5:4:1) at a concentration of 900,000 cells/mL. Till processing the comet assay, samples were stored at −80 °C. The subsequent protocol was identical to that for BEAS-2B cells (Section 2.6.6).

#### 2.7.6. Gene Expression Analysis (RT-qPCR)

EpiDermFT™. Total RNA was extracted using the RNeasy Mini Kit with on-column DNase digestion (Qiagen, Hilden, Germany) according to the manufacturer’s protocol. RNA concentration and purity were determined spectrophotometrically using a NanoDrop 1000 (Thermo Fisher Scientific, Waltham, MA, USA). Complementary DNA (cDNA) was synthesised from 500 ng of total RNA using the qScript/JScript cDNA Synthesis Kit (Quantabio, Beverly, MA, USA). 

Quantitative real-time PCR was performed on a CFX384 thermocycler (Bio-Rad, Hercules, CA, USA) using TaqMan^®^ Gene Expression Master Mix (Applied Biosystems, Foster City, CA, USA) in 10 µL reaction volumes, each run in technical duplicates. Negative template controls (NTCs) were included in all runs. The same gene panel was analysed for both models, targeting markers of (i) inflammation and immune response (*IL-6*, *TNFα*, *PTGS2*), (ii) oxidative stress (*SOD1*, *CAT*, *OGG1*), and (iii) apoptosis/DNA damage (*BCL-2*, *BAX*, *TP53*). Reference genes included *ACTB* (β-actin) and *GAPDH*. The following TaqMan^®^ Gene Expression Assays (Applied Biosystems) were used: Hs99999905_m1 (*GAPDH*), Hs99999903_m1 (*ACTB*), Hs00533490_m1 (*SOD1*), Hs00156308_m1 (*CAT*), Hs00174131_m1 (*IL-6*), Hs00174128_m1 (*TNFα*), Hs01034249_m1 (*TP53*), Hs00153133_m1 (*PTGS2*), Hs00213454_m1 (*OGG1*), Hs00180269_m1 (*BAX*), and Hs99999018_m1 (*BCL-2*). 

The qPCR reactions were carried out under standard cycling conditions recommended for TaqMan^®^ chemistry and analysed using CFX Manager Software v3.1 (Bio-Rad). Gene expression data were normalised to reference genes and analysed using the 2^−ΔΔCt^ method. Results are expressed as Fold Change (FC) relative to control samples exposed to clean synthetic air (MucilAir™) or pure assay medium (EpiDermFT™), with significance determined at *p* < 0.05. All molecular analyses were performed at the GeneCore facility, BIOCEV (Vestec, Czech Republic).

### 2.8. Filtration Testing According to EN 149:2001

The filtration performance of electrospun nanofiber membranes incorporating HBP was evaluated in accordance with EN 149:2001 [49] requirements for filtering materials used in respiratory protective devices. Filtration efficiency tests were performed using a TSI 8130A Automated Filter Tester (TSI Inc., Shoreview, MN, USA) employing a paraffin oil aerosol as the test medium. The generated aerosol had a mean particle diameter of 0.3 µm, corresponding to the most penetrating particle size (MPPS) for fibrous filters. Each sample was tested at a constant volumetric airflow rate of 95 L min^−1^, as defined by the standard, under controlled laboratory conditions (ambient temperature 23 ± 2 °C; relative humidity 30–70%). The instrument simultaneously measured upstream and downstream particle concentrations using a dual-light-scattering photometer, enabling real-time calculation of filtration efficiency (%) and pressure drop (Pa) across the sample. Filtration efficiency (η) was calculated as the percentage reduction in particle concentration between the upstream and downstream sides of the sample, using the equation η (%) = [1 − (C_down/C_up)] × 100, where C_up and C_down represent the upstream and downstream aerosol concentrations, respectively. Each membrane was tested in triplicate to ensure reproducibility. The obtained data were used to assess the impact of hyperbranched polymer type and concentration on the nanofiber structure’s filtration efficiency and air permeability.

### 2.9. Statistics

Statistical evaluation was performed using GraphPad Prism 8. Normality was assessed using the Shapiro–Wilk test at each experiment and time point. If the normality test passed, a One-Way ANOVA with Dunnett’s multiple comparisons test was performed to compare the materials with the control. When the normality test failed, the Kruskal–Wallis test with Dunn’s multiple comparisons was performed. The statistical difference (*p*-value ≤ 0.05) of any material from the control is marked as an asterisk above the column of the material for the specific time point.

## 3. Results

### 3.1. Characterisation of HBPs

**Figure 2:**
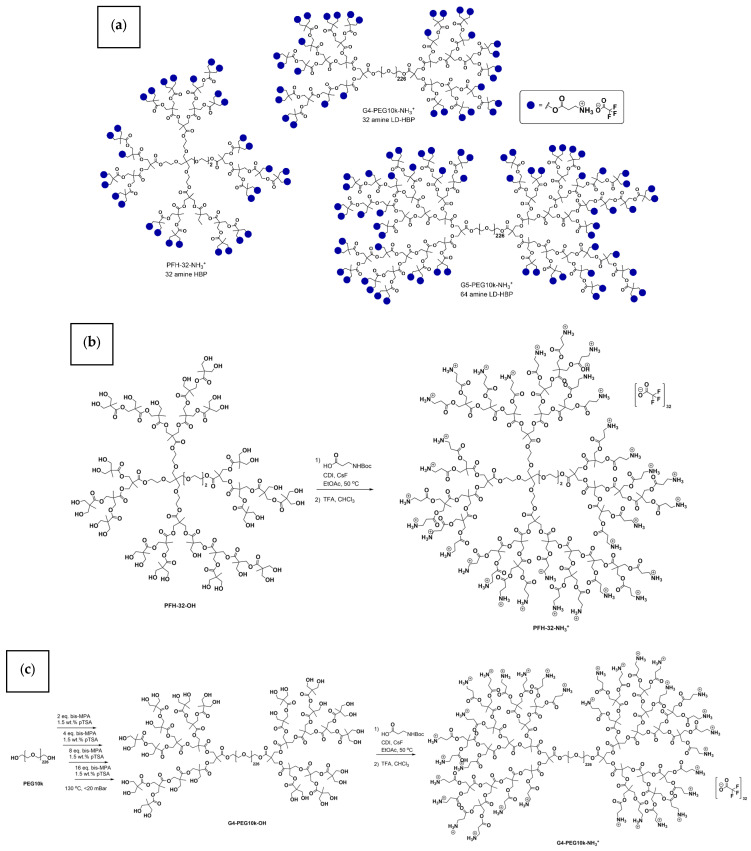

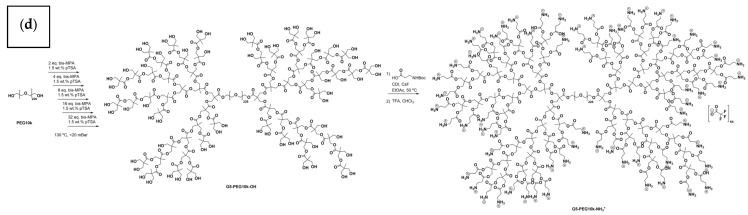
Cationic HBPs were used in this study (**a**) synthesis schematic of PFH-32-NH_3_^+^ (**b**), PFLDHB-G4-PEG10k-NH_3_^+^ (**c**), PFLDHB-G5-PEG10k-OH and PFLDHB-G5-PEG10k-NH_3_^+^ (**d**).

A range of dendritic polymers was produced to serve as antimicrobial additives for electrospun fibers. HBPs are produced by growing polymeric layers, or generations, in which each generation yields exponentially more end groups that can be equipped with chemical groups to impart specific functions. The polymeric architectures chosen were: a globular hyperbranched polymer with 32 amino end groups; a pseudo-generation 4 linear-dendritic hyperbranched polymer (PFH-32, LD-HBP) with 32 amino end groups. (Figure 2). The linear-dendritic hyperbranched structures include a 10 kDa (PFLDHB-G4-PEG10k, PFLDHB-G5-PEG10k) polyethene glycol core to increase compatibility with the fibers’ bulk matrix and improve solubility. A hydroxyl analogue of the generation 5 LD-HBP was also included in the MIC/MBC assays to confirm that any detected antimicrobial activity was due to the cationic amines. The synthesised cationic polymers, their properties, and synthesis schematics are shown in Figure 2.

NMR spectroscopy was employed to confirm the successful synthesis and functionalization of the amine-terminated hyperbranched polymer (PFLDHP-G5-PEG10k-NH_3_^+^). In the ^13^C NMR spectrum, the absence of signals corresponding to methylene carbons adjacent to hydroxyl groups (typically observed at ~60–65 ppm) confirms the complete conversion of hydroxyl functionalities from the precursor polymer. Instead, new ester-related carbonyl signals are observed in the region of ~170–175 ppm, indicating quantitative ester formation [50].

The ^1^H NMR spectrum shows characteristic resonances of the PEG backbone, with broad methylene proton signals centered at ~3.5–3.7 ppm, confirming the integrity of the PEG framework after functionalization. Signals associated with tert-butoxycarbonyl (Boc) protecting groups—previously observed at ~1.4 ppm in ^1^H NMR and ~28 ppm (C(CH_3_)_3_) and ~155 ppm (C=O) in ^13^C NMR—are absent in the final product, demonstrating complete removal of the Boc groups [18]. The appearance of broadened proton resonances associated with protonated amine groups further supports successful deprotection and formation of NH_3_^+^ termini.

**Figure 3:**
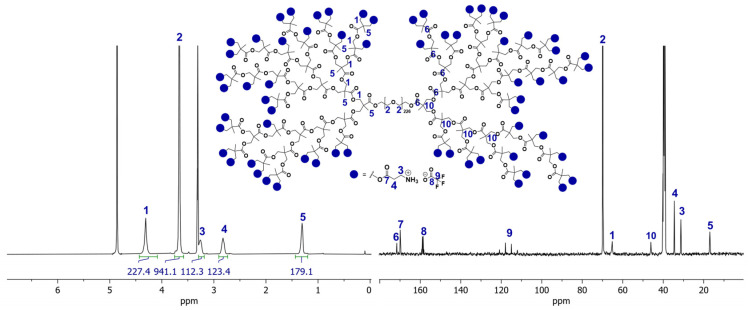
Nuclear magnetic resonance spectra characterisation of HPBs.

Overall, the combined ^1^H and ^13^C NMR spectra confirm (i) full conversion of hydroxyl groups, (ii) quantitative deprotection of amine functionalities, and (iii) preservation of the PEG backbone architecture. The observed peak broadening is consistent with the hyperbranched macromolecular structure and the polymer’s high molecular weight. Detailed spectra and peak assignments for PFLDHP-G5-PEG10k-NH_3_^+^ are provided in Figure 3 below.

The amine-functional HBPs conform well to theoretical values, indicating that the polymers produced have a high density of cationic groups, as postulated to impart antibacterial activity to electrospun nanofibers. NMR molecular mass and number of end groups are shown in Table 2.

### 3.2. Antimicrobial Properties of HBP

To characterise the intrinsic biocidal activity of the various additives studied, Minimum Inhibitory Concentration (MIC) and Minimum Bactericidal Concentration (MBC) of the materials listed in Table 2 were determined. The HBPs chosen for testing represented a range of cationic materials with bactericidal activity but differing polymeric architectures: two linear dendritic materials of different generations and numbers of cationic groups were selected, along with a hyperbranched polymer of generation 3 with a high number of cationic groups per gram. Additionally, a hydroxyl-functional HBP, which is not expected to exhibit antibacterial activity, was tested.

The different products were serially diluted in a growth medium suitable for each challenge microorganism (0.8%, 0.4%, 0.08%, 0.008%). MIC and MBC testing define a test material’s potency in terms of the concentration at which it inhibits growth (MIC) or completely kills (MBC) the challenge microorganisms during an 18-to-20-h incubation period (35 ± 2 °C). Table 3 presents MIC and MBC values for the different products, enabling identification of additives with superior antimicrobial properties.

For *S. aureus*, the polymers generally showed limited activity. Both PFH-32-NH_3_^+^ and PFLDHB-G4-PEG10k-NH_3_^+^ exhibited MIC values above 0.8%, with bactericidal activity observed only at the highest tested concentration (0.8%). In contrast, PFLDHB-G5-PEG10k-NH_3_^+^ showed measurable inhibition at 0.8% and complete bactericidal activity at 0.4%, indicating that higher-generation dendritic structures with cationic groups can more effectively reduce the viability of Gram-positive bacteria. The hydroxyl-terminated polymer PFLDHB-G5-PEG10k-OH displayed no detectable inhibitory or bactericidal effect within the concentration range studied. Against *E. coli*, markedly higher sensitivity was observed. PFH-32-NH_3_^+^ demonstrated the strongest activity, with inhibition at 0.08% and complete killing at only 0.008%, representing the lowest MBC value in this study. PFLDHB-G4-PEG10k-NH_3_^+^ inhibited growth at 0.4% and was bactericidal at 0.08%, whereas PFLDHB-G5-PEG10k-NH_3_^+^ showed weaker performance, with both MIC and MBC values shifted to 0.8% and 0.4%, respectively. As expected, PFLDHB-G5-PEG10k-OH did not exhibit a low MIC or MBC, confirming that the polymers’ antibacterial activity is due to the terminal cationic groups.

The reduced antibacterial efficacy observed against *S. aureus*, compared with *E. coli*, is attributable to the resilient, highly crosslinked peptidoglycan layer characteristic of Gram-positive bacteria; this structure impedes the infiltration and membrane accessibility of hyperbranched polymers. Conversely, the negatively charged and less rigid outer membrane of Gram-negative *E. coli* facilitates electrostatic interactions, thereby promoting more effective contact-active membrane disruption [51].

### 3.3. Electrospinning of Nanofibers with HBPs

**Figure 4:**
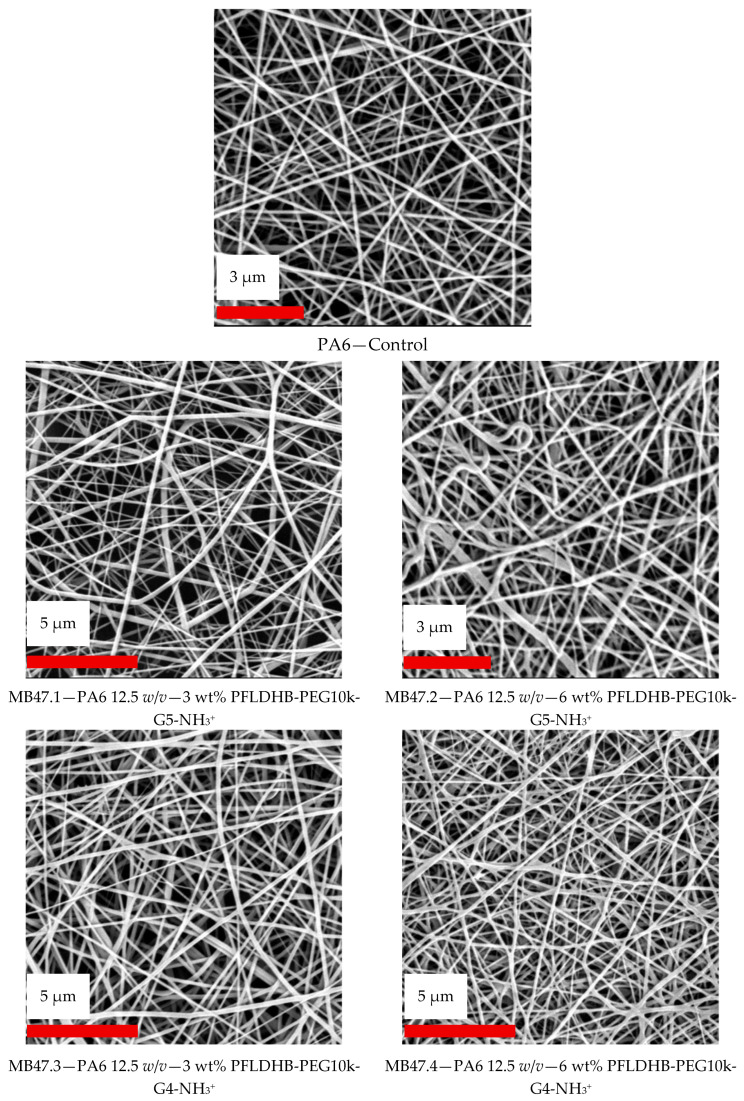
Morphology of pristine PA6 and hybrid PA6/HBP nanofibers.

Based on the results of antimicrobial property screening of HBPs, we have selected PFLDHB-G4-PEG10k-NH_3_^+^ and PFLDHB-G5-PEG10k-NH_3_^+^ for incorporation into nanofibers (Table 4). Polymeric solutions containing 3% and 6% HBPs were prepared by needleless electrospinning with 12.5% PA6 as the carrier matrix. The electrospinning experiment showed successful formation of nanofibers. The morphology analysis shows smooth fiber formation, free of defects or beads (Figure 4).

The pristine PA6 control nanofibers, prepared without the addition of any HBP, exhibited relatively thin fibers with an average diameter of approximately 120 nm and a mean pore size of 0.72 ± 0.14 µm. In contrast, the incorporation of HBP into the PA6 spinning solution increased solution viscosity, resulting in a noticeable increase in the average nanofiber diameter [52]. The MB47.1 sample (3% of PFLDHB-G5-PEG10k-NH_3_^+^) showed a mean fiber diameter of 420 ± 220 nm, a mean pore size of 1.08 ± 0.70 µm, and apparent porosity of 60.4 ± 5.60%. The sample MB47.2 (6% of PFLDHB-G5-PEG10k-NH_3_^+^) showed a mean fiber diameter of 520 ± 240 nm, a mean pore size of 1.37 ± 0.75 µm, and apparent porosity of 49.6 ± 3.40%. The sample MB47.3 (3% of PFLDHB-G4-PEG10k-NH_3_^+^) showed a mean fiber diameter of 410 ± 180 nm, a mean pore size of 1.10 ± 0.54 µm, and an apparent porosity of 63.8 ± 8.29%. Finally, the sample MB47.4 (6% of PFLDHB-G4-PEG10k-NH_3_^+^) showed a mean fiber diameter of 470 ± 200 nm, a mean pore size of 1.22 ± 0.35 µm, and an apparent porosity of 49.4 ± 5.31%. The results indicate that nanofiber diameter depends on HBP loading. With increased HBP concentration, the fiber diameter and porosity increase, as observed in electrospinning scaling rules.

**Figure 5:**
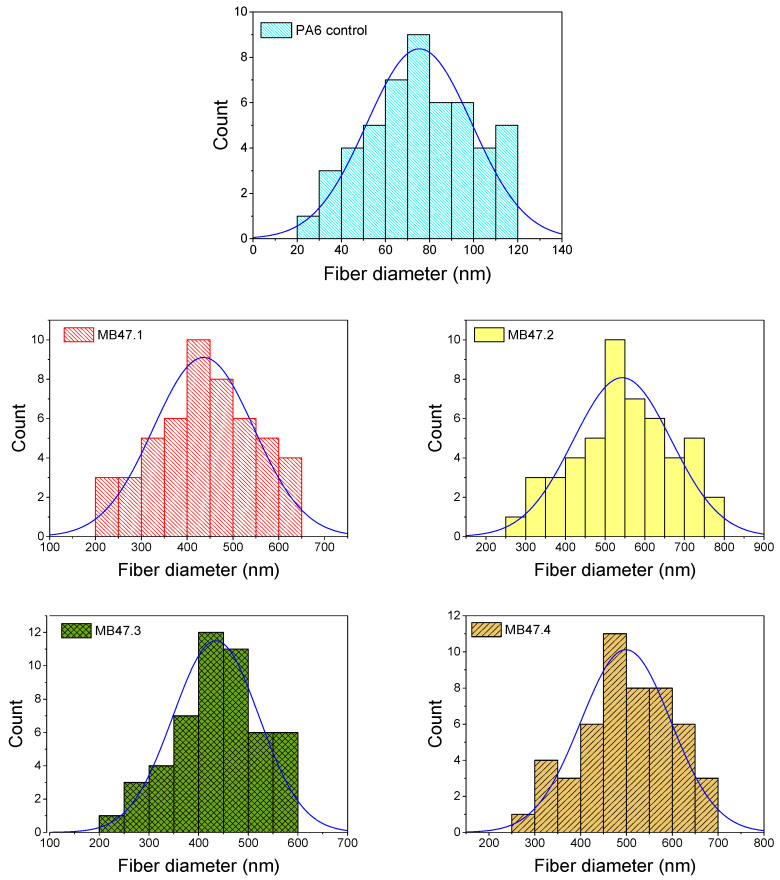
Fiber diameter distribution histogram of pristine PA6 and hybrid PA6/HBP nanofibers.

The fiber diameter distribution histograms in Figure 5, derived from 50 individual fiber diameter measurements, indicate that pristine PA6 produced relatively homogeneous nanofibers with a narrow, unimodal distribution centered at ~120 nm, suggesting stable jet thinning and constant stretching. The introduction of HBP shifted the distributions towards significantly larger diameters (approximately 410–520 nm) and significantly increased polydispersity, consistent with viscosity/viscoelasticity-induced suppression of jet thinning and heightened variability in electrohydrodynamic stretching [37]. Augmenting HBP loading from 3 wt.% to 6 wt.% resulted in an additional rightward shift in the diameter distributions, while preserving wide variances, indicating heterogeneous local stretching circumstances throughout the spinning process. The expanded diameter distributions are associated with an increased mean pore size [53]; yet, apparent porosity diminishes with higher HBP loading, indicating a denser mat structure despite bigger characteristic pore openings.

**Figure 6:**
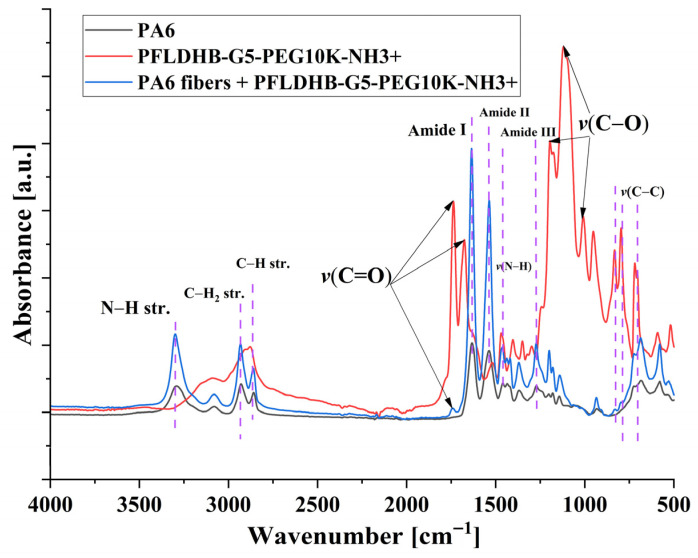
ATR-FTIR analysis of the fibers showing typical absorbance peaks corresponding to the HBPs and PA polymer.

The ATR-FTIR spectra of the samples showed characteristic absorbance peaks for the bulk polymer and the HBPs (Figure 6). An N-H stretching was observed for the bulk polymer and the functionalized fibers at 3300 cm^−1^. Further absorbance peaks associated with the asymmetric stretching of C-H_2_ and C-H were observed in all the measured samples [54]. Expectedly, strong νC=O vibration absorption peaks were observed for the HBPs at 1741 cm^−1^ and 1685 cm^−1^ [55]. However, functionalized fiber samples demonstrated only a single νC=O vibration absorption peak at 1741 cm^−1^. The amide I, II, and III peaks were measured at 1640 cm^−1^, 1540 cm^−1^, and 1450 cm^−1^ for the bulk polymer and functionalized fibers, respectively. Noticeably, all the samples demonstrated a primary amine bend absorbance peak at 1680 cm^−1^ [56].

Corresponding C-O vibration absorbance peaks were observed between 1260–1010 cm^−1^ strongly for HBP samples and weakly for functionalized fibers. Overall, the FTIR spectra of the fibers closely match those of the bulk polymer. However, some characteristic peaks of HBPs were observed, demonstrating their presence in functionalized fibers.

### 3.4. Antimicrobiological Characterisation of HBP Samples

The antibacterial performance of the hyperbranched polymer HBP-modified nanofibers was further evaluated against *Staphylococcus aureus* subsp. *Aureus* CCM 2022 using a quantitative plate count assay. The initial inoculum in all experiments was standardised to 1.16 × 10^8^ CFU/mL. Electrospun PA6 nanofiber mats containing 6 wt.% of either PFLDHB-G5-PEG10k-NH_3_^+^ (sample MB47.2) or PFLDHB-G4-PEG10k-NH_3_^+^ (sample MB47.4) were coincubated for 2 h in phosphate buffer with the bacterial suspension, after which viable counts were determined by the Plate Count Agar method (Table 5).

**Figure 7:**
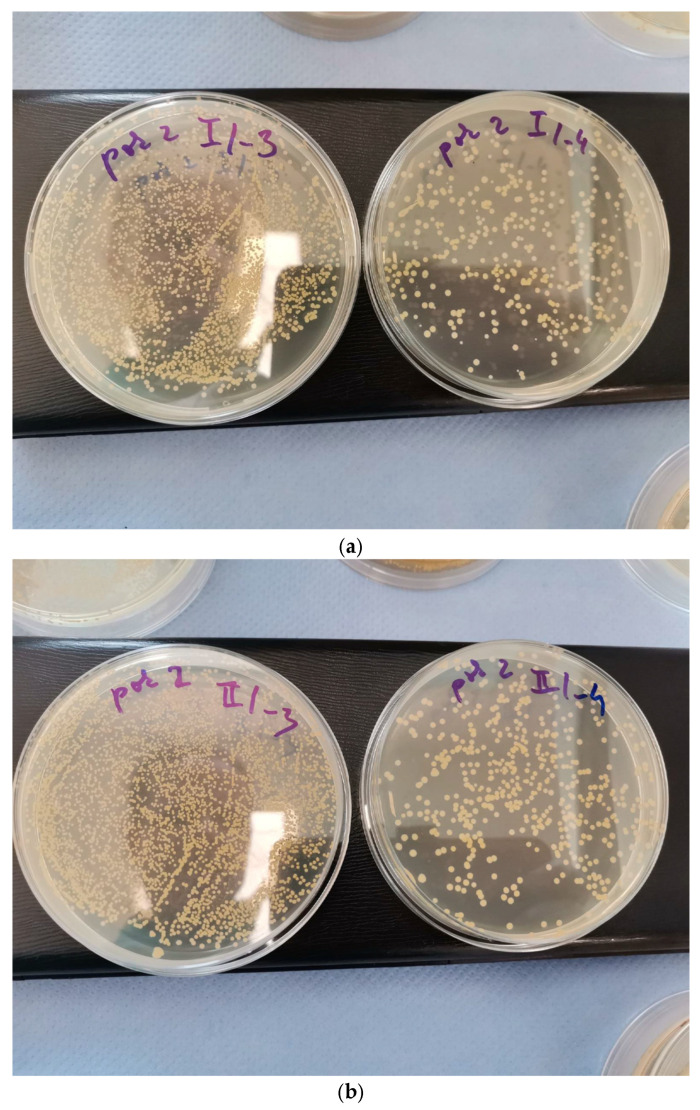
Coincubation of nanomaterials, **Left**: Suspension with coincubated nanomaterial diluted 3 times, **Right**: Suspension with coincubated nanomaterial diluted 4 times. (**a**) MB47.2, (**b**) MB47.4.

The results demonstrated an apparent antibacterial effect for both formulations, with differences reflecting the polymer generation (Table 5, Figure 7). For MB47.2 (PA6 + 6 wt.% PFLDHB-G5–PEG10k-NH_3_^+^), the viable bacterial concentration decreased from 1.16 × 10^8^ CFU/mL to 3.0 × 10^7^ CFU/mL after 2 h, corresponding to a reduction of 74.1%. Since only the liquid phase was analyzed, this reduction primarily indicates bactericidal or bacteriostatic activity induced by contact exposure, rather than bacterial adhesion inhibition. Time-dependent kill kinetics were not evaluated in the present study; however, the selected 2 h endpoint provides a standardized comparison with the diffusion assay under identical bacterial loading conditions. In contrast, MB47.4 (PA6 + 6 wt.% PFLDHB-G4–PEG10k-NH_3_^+^) resulted in a decrease from 1.16 × 10^8^ CFU/mL to 5.08 × 10^7^ CFU/mL, corresponding to a 56.2% reduction in bacteria.

These findings are consistent with MIC/MBC assays, in which cationic G5 derivatives showed greater activity than G4 analogues. The enhanced efficacy of the G5-modified nanofibers can be attributed to the higher density of terminal amine groups, which promote stronger electrostatic interactions with the negatively charged bacterial cell envelope, ultimately leading to membrane disruption and loss of viability [57].

The antibacterial activity of the nanofiber mats was also examined using an adaptation of ISO 20645:2005, which measures the diffusion of active agents through agar to form inhibition zones around the tested material and also evaluates growth on the sample surface. Samples MB47.2 (PA6 with 6 wt.% PFLDHB-G5-PEG10k-NH_3_^+^) and MB47.4 (PA6 with 6 wt.% PFLDHB-G4-PEG10k-NH_3_^+^) were evaluated against *Staphylococcus aureus* and *Escherichia coli*. In all cases, including the PA6 control, no measurable inhibition zones were detected. Importantly, however, no bacterial growth was observed directly underneath the nanofiber samples. This result likely indicates that the samples exhibit a significant antimicrobial effect due to the presence of HBP, and that HBP does not spread beyond the samples. 

This outcome indicates that the antimicrobial activity of the hyperbranched polymer-modified fibers is primarily contact-mediated. Because cationic HBPs are stably incorporated into the nanofiber matrix and exhibit minimal leaching into the surrounding medium, their antimicrobial activity does not extend into the agar [58,59]. It thus cannot produce a halo of inhibition. Instead, bacterial viability reduction occurs exclusively at the fiber–cell interface, consistent with a mechanism based on electrostatic interactions and membrane disruption upon direct contact. Taken together with the CFU reduction assays, these results confirm that HBP-functionalized nanofibers possess significant antimicrobial potential, but their mode of action is surface-restricted rather than diffusion-mediated.

### 3.5. Evaluation of Biocompatibility and Cellular Toxicity on In Vitro Cell Cultures

To evaluate the safety profile of the nanofiber–HBP system, we have conducted in-depth toxicological testing using relevant cell lines. The routes of exposure include topical contact with skin cells and the upper airway route. The key rationale is the potential for long-term direct contact, and thus the possibility of leaching or emission of substances from the nanosystem onto the skin or into the lungs, upon use of the nanosystem in personal protective equipment (PPE) for the general public.

The cytocompatibility of nanofiber composites MB47.2 and MB47.4 was assessed using a comprehensive panel of assays across mouse fibroblasts (3T3), human keratinocytes (HaCaT), murine melanocytes (Melan-a), and human bronchial epithelial cells (BEAS-2B). 24-h extracts prepared from both materials were applied to cell cultures and incubated for 24 and 72 h. The culture medium served as a negative control.

**Figure 8:**
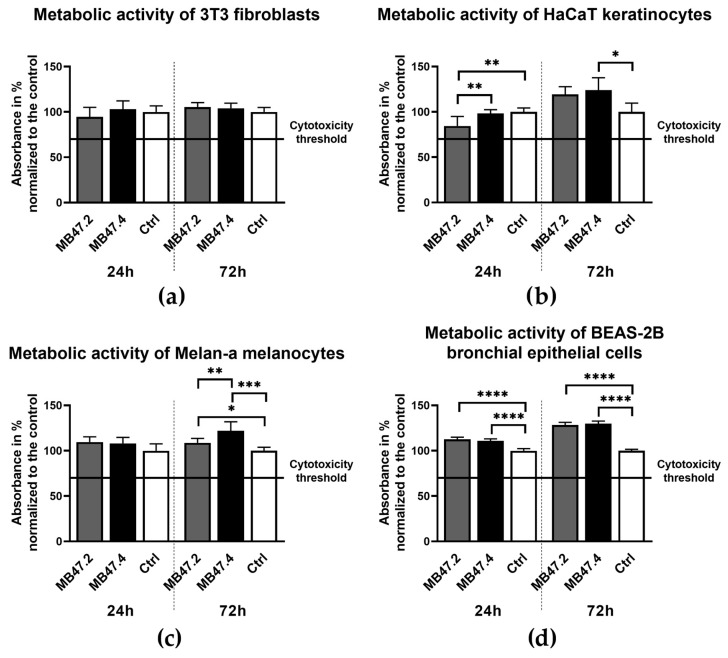
Metabolic activity of (**a**) 3T3 fibroblasts, (**b**) HaCaT keratinocytes, (**c**) Melan-a melanocytes, and (**d**) BEAS-2B bronchial epithelial cells in 24-h extracts from MB47.2 and MB47.4 materials plotted in absorbance of MTS test normalized to the control group. Culture medium served as the negative control (100%). Metabolic activity was analysed at 24 and 72 h after exposure to the extracts. The cytotoxicity threshold is 70% of the control. The statistical difference between the groups is marked as a line. * *p* < 0.05, ** *p* < 0.01, *** *p* < 0.001, **** *p* < 0.0001.

The potential cytotoxicity was assessed by quantifying metabolic activity using the MTS assay (Figure 8). Across all cell types, values remained well above the cytotoxicity threshold (70% of the negative control) defined by ISO 10993-5 [60]. The metabolic activity of 3T3 fibroblasts in extracts from MB47.2, MB47.4, and control culture medium was comparable for both time intervals. HaCaT keratinocytes displayed lower values in MB47.2 extract at 24 h and higher values in MB47.4 extract compared to the control at 72 h. Melan-a melanocytes showed results comparable to the control at 24 h. The highest metabolic activity was observed in the MB47.4 extract at 72 h, and the MB47.2 extract showed higher activity than the control. BEAS-2B bronchial epithelial cells showed increased metabolic activity compared to the control in both extracts at both time intervals.

**Figure 9:**
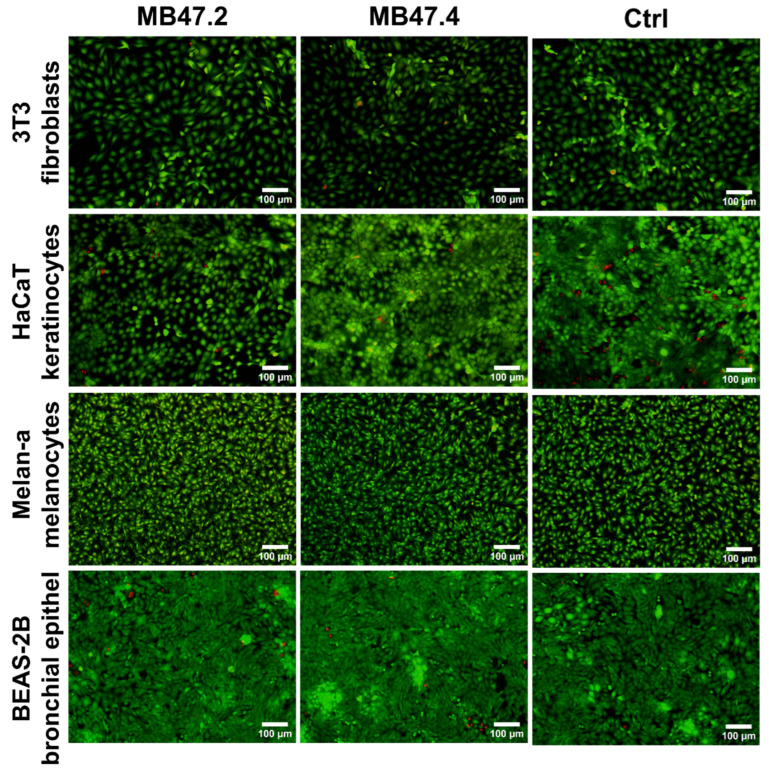
Live/Dead staining of 3T3 fibroblasts, HaCaT keratinocytes, Melan-a melanocytes, and BEAS-2B bronchial epithelial cells cultured for 72 h in extracts from MB47.2 and MB47.4. Culture medium served as a negative control. Cytoplasm of live cells stained in green, nucleus of dead cells stained in red. Scale bar 100 µm.

Live/Dead staining distinguishes live (green cytoplasm) from dead (red nucleus) cells. The pictures confirmed the data obtained by metabolic activity and proliferation measurements (Figure 9). No cytotoxicity was observed; nearly no dead cells were visible, cells showed a spread morphology, and the number increased with incubation time.

**Figure 10:**
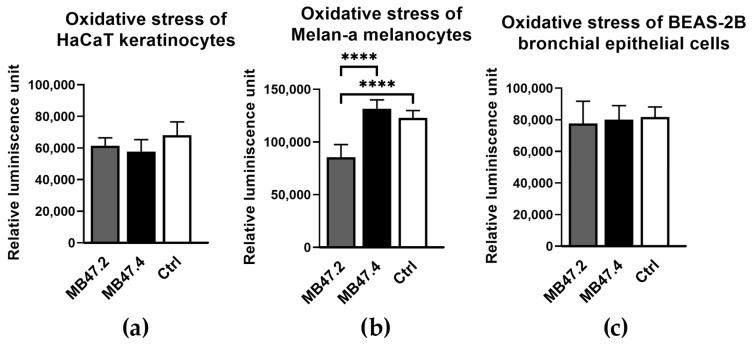
Oxidative stress was measured as the produced H_2_O_2_ in the culture of (**a**) HaCaT keratinocytes, (**b**) Melan-a melanocytes, and (**c**) BEAS-2B bronchial epithelial cells cultivated in extracts from MB47.2 and MB47.4 materials for 1.5 h. Culture medium served as a negative control. A positive control confirmed the essay’s functionality, the data are in Supplementary data (S1). The statistical difference between the groups is marked as a line. **** *p* < 0.0001.

Oxidative stress, analysed 1.5 h after the exposure, was measured only for the more sensitive cells, so 3T3 fibroblasts were excluded. No increased reactive oxygen species (ROS) production was observed compared to the control, meaning that the materials did not exhibit higher levels of oxidative stress (Figure 10). In contrast, lower ROS production was observed in MB47.2 extract compared to MB47.4 extract and the control culture medium.

**Figure 11:**
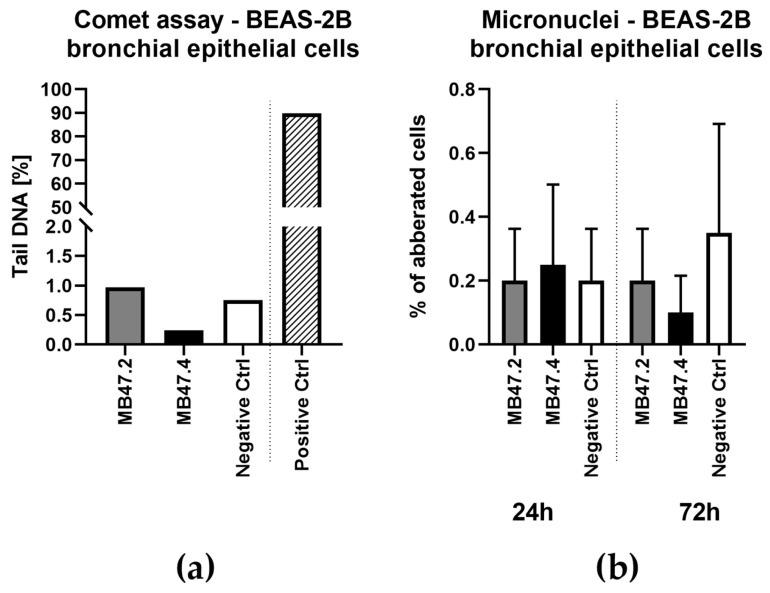
(**a**) Comet assay and (**b**) micronuclei assay analysed on BEAS-2B bronchial epithelial cells 24 h, in the case of the comet assay, or 24 and 72 h, in the case of the micronuclei assay, after the exposure.

Both the comet and micronucleus assays excluded genotoxicity. Materials showed levels of oxidative stress comparable to those of the negative control and, in the comet assay, much lower values than those of the positive control (Figure 11).

### 3.6. Evaluation of Biocompatibility Using Reconstructed Human Skin and Upper Airway Models

**Figure 12:**
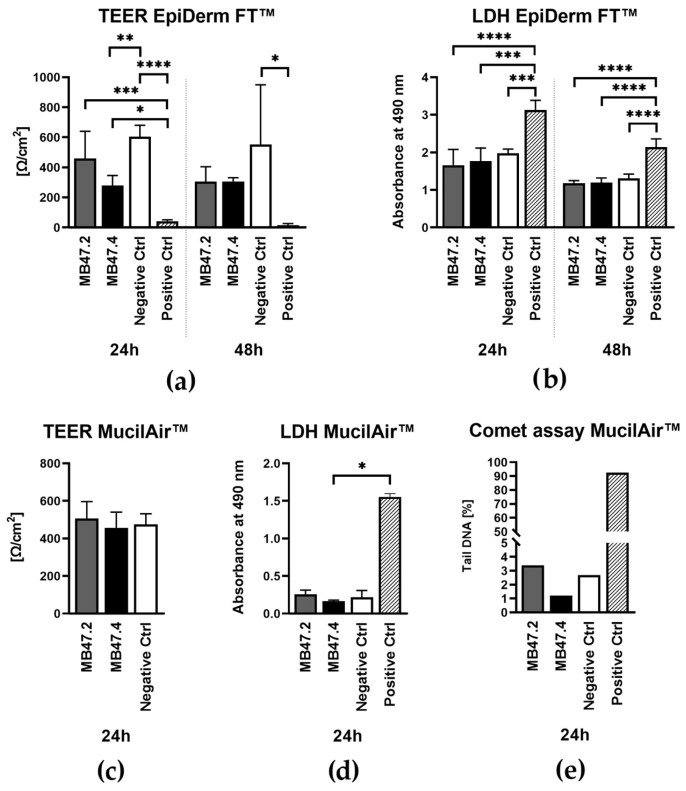
Measurement of (**a**) transepithelial electrical resistance (TEER) and (**b**) lactate dehydrogenase (LDH) on EpiDerm FT™ models incubated with extracts from MB47.2 and MB47.4 for 24 and 48 h. And measurement of (**c**) TEER, (**d**) LDH, and (**e**) comet assay on MucilAir™ models, which were exposed to the air flow through the materials MB47.2 and MB47.4 in a specially designed chamber, 24 h after the exposure. * *p* < 0.05, ** *p* < 0.01, *** *p* < 0.001, **** *p* < 0.0001.

The potential toxicity of MB47.2 and MB47.4 was examined in tissue models: EpiDerm FT™ as an extract, and MucilAir™ as airflow passing through the sample. The transepithelial electrical resistance (TEER) measured on EpiDerm FT™ showed lower values of MB47.4 than the negative control. Still, the positive control showed an even lower value, which was significantly lower than those of all the tested groups after 24 h of exposure (Figure 12). 72-h exposure showed comparable values for both materials relative to the negative control. However, only the negative control showed a significant difference from the positive control. Lactate dehydrogenase (LDH) demonstrated a favourable biocompatibility of the materials on the EpiDermFT™ model. LDH release was comparable to the negative control and significantly lower than the positive control on both tested time intervals.

Under dynamic exposure conditions [47,48] simulating respiratory airflow, nanofiber membranes MB47.2 and MB47.4 were evaluated using the MucilAir™ 3D human airway epithelial model and compared to synthetic air without added contaminants. The TEER was comparable across both materials and the negative control, indicating that the cell layer was not impaired (Figure 12). LDH activity measured in the culture medium confirmed the absence of cytotoxicity, as the materials showed values comparable to those of the negative control. The positive control showed visibly higher values, though the statistical difference was proven only for MB47.4. The comet assay also showed very low levels of oxidative damage caused by the materials compared to the positive control.

Gene expression profiling targeting immunological *(IL6*, *TNF*, *PTGS2*), oxidative stress (*SOD1*, *CAT*, *OGG1*), and apoptotic (*TP53*, *BCL2*, *BAX*) markers revealed that neither MB47.2 nor MB47.4 upregulated expression of any of these genes in EpiDerm FT™ (Table 6). Downregulation of oxidative stress (*OGG1*) and apoptotic (*BAX*) markers further confirmed an absence of oxidative or cytotoxic cellular responses. Collectively, MB47.2 exhibited a consistently stable cellular profile, while MB47.4 showed transient tight junction disruption without lasting cytotoxic or pro-inflammatory effects.

Gene expression analysis in MucilAir™ revealed upregulation (fold change [FC] > 1) of all three oxidative stress markers in the case of exposure to MB47.2, indicating a mild oxidative response without downstream activation of apoptotic pathways (Table 6). Expression levels of *IL-6* and *PTGS2* were downregulated (fold change [FC] < 1) following exposure to MB47.2 (both genes) and MB47.4 (*IL-6* only), suggesting an absence of inflammatory response. Overall, both MB47.2 and MB47.4 nanofiber membranes were well tolerated by the airway epithelium, showing no cytotoxic, inflammatory, or barrier-compromising effects.

### 3.7. Evaluation of Filtration Efficacy

Filtration performance of the electrospun nanofiber membranes incorporating dendritic hyperbranched polymers (HBPs) was assessed using a TSI 8130A Automated Filter Tester (TSI Inc., Shoreview, MN, USA) in accordance with EN 149:2001. Tests were conducted with a paraffin oil aerosol (mean particle diameter 0.3 µm) at an airflow rate of 95 L min^−1^, corresponding to the most penetrating particle size and operational flow conditions defined by the standard. Each membrane sample was analysed in triplicate to determine filtration efficiency (%) and pressure drop (mm H_2_O), as summarised in Table 7. All tested nanofiber membranes exhibited exceptionally high filtration efficiencies, consistently exceeding 99.8%, thereby fulfilling and surpassing the EN 149:2001 FFP3 classification threshold (≥99%). The highest performance was observed for MB47.1 (PA6 + 3% PEG-10k-G5-NH_3_^+^), achieving 99.995–99.998% removal efficiency across replicates. Membranes containing generation 5 (G5) HBPs demonstrated marginally superior efficiencies compared with generation 4 (G4) analogues, suggesting that higher dendritic branching enhances fiber surface area and electrostatic capture potential.

The recorded pressure drop values ranged from 36 to 57 mm H_2_O, which are typical for compact electrospun layers at this test flow rate and remain compatible with respirator-grade materials. The lowest pressure drop was noted for MB47.2, indicating improved air permeability at elevated HBP content (6%). All samples exhibited stable mechanical integrity and consistent aerosol retention performance across repeated measurements. These results confirm that PA6-based nanofiber membranes functionalized with PEG-modified dendritic polymers provide FFP3-level particle filtration efficiency while maintaining acceptable breathing resistance.

## 4. Discussion

### 4.1. Public Health Motivation: Pandemics and Air-Pollution Mitigation

The COVID-19 pandemic has underscored the need for advanced personal protective equipment as part of future pandemic preparedness [61,62]. The importance of such measures is highlighted by pandemic analyses that note how pandemics tend to recur over time. History has witnessed multiple pandemic cycles, such as the 1918 “Spanish Flu”, SARS in 2002, swine flu in 2009, and the 2019 COVID-19 outbreaks. Experts are warning that novel respiratory viruses will emerge again and may cause new pandemics affecting lives and economies around the globe [63]. Grey and Abdelgadir [64] similarly stress the importance of developing advanced filtration materials as an investment for increased resilience for future pandemics.

Beyond infectious diseases, the broad health benefits of effective air filtration are increasingly recognised. Air pollution remains a significant global health burden, contributing to millions of premature deaths annually [65]. Fine particulate matter (PM_2.5_) in particular is linked to cardiovascular and respiratory diseases [65,66]. Personal respiratory filters and face masks can mitigate these risks by reducing the dose of inhaled pollutants. A recent large-scale study in Delhi demonstrated that routine mask use during severe smog episodes could prevent a significant fraction of pollution-related mortality and morbidity [67]. Wearing high-efficiency masks during peak pollution days was estimated to avert ~13% of short-term excess deaths, yielding substantial economic benefits from avoided health costs. Such data illustrate that encouraging mask or filter use in polluted environments can measurably improve public health outcomes. Even in controlled experiments, high-performance filter materials have been shown to improve physiological markers. Wearing efficient PM_2.5_ filters can attenuate the rise in blood pressure and inflammatory markers that typically accompany exposure to polluted air. The results of these studies show the potential of advanced air filters to deliver dual health benefits by reducing exposure to infectious bioaerosols and harmful pollutants [68].

### 4.2. Sustainability Challenge: Disposable PPE Waste and the Need for Reusable/Self-Disinfecting Filters

Disposable masks and respirators are responsible for an unprecedented surge in plastic medical waste [69]. Conventional polypropylene masks are not biodegradable, straining waste management and causing environmental challenges. Recent reviews emphasise that current mask disposal methods are unsustainable and call for innovative solutions to reduce PPE waste. The utilisation of biodegradable polymers is delivering a sustainable pathway. However, due to its short lifespan and complex composting procedures, it is associated with high waste generation and an incomplete waste-disposal strategy [70]. As an alternative, reusable, self-disinfecting air filters could help address this issue by extending product life and reducing waste. In particular, filters that can inactivate pathogens on contact offer a dual benefit: they remain safer for reuse (diminishing infectious waste) and continuously protect users.

### 4.3. Key Innovation: Recycled-PA6, Green Solvent Electrospinning, and FFP3-Grade Performance at Low Pressure Drop

The current study aimed to develop an antimicrobial self-cleaning respiratory membrane that enables prolonged use time and reduces the risk of pathogen proliferation on the membrane, thereby reducing the risk of contamination. Technically, the solution is based on a combination of antimicrobial hyperbranched dendritic polymers with suitable polymers for the production of nanofibers. In this case, we have utilised polyamide 6 from recycled sources. The formulation was optimised to enable production using green solvents, such as acetic acid and formic acid. Thanks to the recyclability of polyamide 6, the system delivers a green alternative to polypropylene spundbond materials. The nanofiber filter developed in this study exhibited outstanding mechanical filtration performance. Our results showed >99% filtration efficiency for sub-micron particles, meeting the stringent EN 149 FFP3 standard. Importantly, this high capture efficiency was achieved with low air resistance, indicating excellent breathability. The measured pressure drop of our filters was well below the 300 Pa limit for FFP3 respirators, making them comfortable for continuous use. This combination of FFP3-level efficacy and high breathability is a key innovation, since typical high-efficiency respirators often suffer from high airflow resistance. Notably, our electrospun HBP-infused membranes approached the air resistance of surgical masks, while filtering as well as, or better than, commercial FFP3 media. These findings are consistent with other nanofiber-based filters reported in the literature. For instance, Sun et al. achieved ~94–97% filtration efficiency using silver-coated glass fiber filters, with pressure drops of only ~100–150 Pa [71]. Similarly, Li et al. [72] reported that a cross-linked nanofibrous membrane could achieve 99.9% filtration efficiency at a resistance of just 43 Pa. In our case, the integration of a hyperbranched polymer did not compromise the fiber mat’s pore structure or permeability. The electrospun fibers form a porous, high-surface-area network that mechanically sieves particles and also leverages interception and electrostatic mechanisms common to nanofiber filters. Achieving FFP3 filtration ratings with such low resistance is particularly significant for applications like personal protective equipment, where user compliance often hinges on comfort.

### 4.4. Structure–Property Rationale: PA6–HBP Compatibility and Amine-Density Driven Bioactivity

The PEG10k-NH_3_^+^ hyperbranched polymer and PA6 nanofibers interact via strong noncovalent intermolecular forces due to their complementary chemical functionalities. Polar amide groups (–CONH–) on the backbone of PA6 donate and accept hydrogen bonds. In contrast, PEG10k-NH_3_^+^ has a branched poly(ethylene glycol) architecture with protonated amine groups, offering abundant cationic and hydrogen-bond-active sites. Hydrogen bonding occurs between PA6 amide groups’ C=O and PEG-based HBP’s –NH_3_^+^ termini during solution blending and electrospinning. Meanwhile, the PEG backbone’s ether oxygen atoms (–C–O–C–) interact with PA6’s N-H groups as hydrogen bond acceptors. Multiple interaction pathways ensure PEG10k-NH_3_^+^ dispersion in the PA6 nanofiber matrix is compatible and uniform. MIC/MBC assays showed that cationic G5 derivatives were more active than G4 analogues. The higher terminal amine group density of G5-modified nanofibers promotes stronger electrostatic interactions with the negatively charged bacterial cell envelope, leading to membrane disruption and loss of viability.

### 4.5. Antibacterial Mechanism: Contact-Active, Non-Diffusion-Mediated Killing

In addition to passive particle capture, the developed filter exhibits active antimicrobial properties due to the HBP biocide embedded in the fibers. In the antibacterial tests, the HBP-functionalized nanofiber filters exhibited pronounced antibacterial activity against both *Staphylococcus aureus* and *Escherichia coli*. Quantitative plate count assays demonstrated reductions of up to 74% in viable bacterial counts after 2 h of coincubation, confirming that cationic hyperbranched polymers effectively disrupt bacterial membranes via electrostatic interactions. No inhibition zones were observed in agar diffusion assays. At the same time, bacterial growth was absent directly beneath the tested samples, indicating that the antibacterial effect is contact-active and not mediated by diffusible species. This confirms that the polymers are stably embedded within the nanofiber matrix and do not leach into the surrounding environment. No antiviral assays were performed within this study. However, due to their submicron fiber diameter and interconnected pore structure, the electrospun nanofibers are capable of mechanically capturing virus-sized aerosols (typically 80–200 nm) via interception and Brownian diffusion, as shown by filtration efficacy testing. Captured viral particles remain immobilised on the filter surface and cannot replicate in the absence of living host cells, eliminating the risk of proliferation. Thus, while the system contributes to physical removal of viral aerosols from airflow, its demonstrated biological activity is limited to antibacterial effects.

### 4.6. Positioning Versus Literature and Commercial Media: Polymer-Only Antimicrobial Filters

These findings align with prior reports on polymer-based, metal- and antibiotic-free self-sterilising filter media. Jiang et al. [73] spray-coated commercial media with quaternary ammonium salts and demonstrated antimicrobial control of bioaerosols on air filters, highlighting a practical, polymer-only route for respiratory protection. Kim et al. [74] functionalized HEPA fabrics with tannic acid (a polyphenolic polymer coating). They demonstrated markedly enhanced capture of influenza virus in the absence of metals/antibiotics, supporting polymer-surface strategies that immobilise pathogens on filters. For contact-active cationic polymers, Zhou et al. [75] grafted quaternary-ammonium groups onto membranes. They achieved strong bactericidal performance via surface-bound charge—an approach directly translatable to fibrous filter substrates. In addition, polymeric N-halamine modifications of polypropylene nonwovens (no metals/antibiotics) have repeatedly shown rapid kill of airborne bacteria on mask/filter media—first demonstrated by Demir et al. [76]. Moreover, commercial air filter media were provided in Table 8. Commercial facemasks in Table 8 are typically fabricated from melt-blown polypropylene electret nonwovens, which provide high filtration efficiency with a reasonable pressure drop due to the combined effects of mechanical capture and electrostatic attraction. However, these materials are generally biologically inert: they mainly trap microbe-laden aerosols rather than inactivate them, and contamination, moisture loading, or charge decay can limit safe reuse [77]. Consequently, most PP electret masks are treated as single-use items, and their large-scale disposal contributes substantially to plastic waste and microplastic pollution [78].

The present results indicate that embedding hyperbranched dendritic polymers in electrospun nanofibers yields an efficient antibacterial surface that suppresses microbial growth on filters. The system therefore prevents biofilm formation and secondary contamination during prolonged use. Hyperbranched and dendritic polymers are emerging as a class of antimicrobial agents that mimic antimicrobial peptides in disrupting microbial membranes [23]. For example, hyperbranched polylysine nanopolymers were recently shown to inhibit SARS-CoV-2 replication in vitro with low cytotoxicity [84]. Our findings corroborate the notion that nanoscale cationic polymers can effectively inactivate viruses by electrostatic binding and membrane disruption. However, as with some prior systems, the antiviral efficacy of our HBP could benefit from further optimisation—for instance, increasing the density of cationic functional groups or incorporating light-activated moieties to accelerate viral destruction.

### 4.7. Safety-by-Design and Biocompatibility: Minimal Leaching, Low Cytotoxicity, and Advanced Tissue Models

An important advantage of the developed HBP-based approach is its favourable safety and sustainability profile compared with traditional antimicrobial additives such as metal ions or leaching biocides. The hyperbranched polymer used here is covalently incorporated into the nanofibers, minimising leaching into the environment or onto the skin. Unlike silver or copper nanoparticle coatings, which can release metal ions over time and raise concerns about toxicity or environmental persistence, the polymeric biocide is a large, non-volatile molecule designed to remain firmly attached to the filter. This aligns with SSbD design principles, which prioritise materials that are effective yet inherently safer for people and the planet [85]. In terms of chemical safety, our HBP contains no heavy metals or halogenated compounds; it was synthesised from amine-rich monomers that were thoroughly washed of any unreacted species. The final fiber mats showed negligible toxicity in comprehensive biological assays. We conducted cytotoxicity tests with multiple human cell lines representing likely contact tissues—human dermal fibroblasts, keratinocytes, and melanocytes (to model skin exposure to mask/filter materials)—and with human bronchial epithelial cells (BEAS-2B) to model inhalation exposure in the lungs. In all cases, cells cultured in extract media from HBP-infused filters showed viability comparable to that of controls (cell viability remained >90% in all standard assays). There were no signs of cell membrane damage or inflammatory cytokine release attributable to the filter material. These results are consistent with reports that many dendritic and hyperbranched polymers can be engineered for biocompatibility [86].

Furthermore, we evaluated the material on advanced 3D tissue models to simulate real-world exposure scenarios. In a fully differentiated mucociliary respiratory epithelium model (MucilAir^TM^), the developed nanofibers did not impair tissue viability after air exposure, indicating that they are non-irritant to airway mucosa. The only significant effect was an increase in oxidative stress markers (*SOD*, *CAT*, *OGG1*) detected after the exposure of MucilAir^TM^ to MB47.2. Oxidative stress is a compendious process that arises as a consequence of an imbalance between the levels of ROS (reactive oxygen species) and antioxidant defenses [87]. Cells are protected against oxidative damage by the activities of antioxidant enzymes and other molecules [88]. If the capacity of antioxidant mechanisms is insufficient, ROS induce processes leading to activation of transcription factors resulting in release of pro-inflammatory cytokines [89]. Based on the significant decrease in these molecules (*IL-6* and *PTGS2*) in our study, we believe that the antioxidant capacity of the cells is satisfactory. Exposure to this material does not induce any adverse effects linked to oxidative stress. Likewise, in a full-thickness skin model (Epiderm FT^TM^), no significant irritation or toxicity was observed upon exposure to the filtering material. These results provide confidence that the filter can be worn against the skin and breathed through safely, without causing dermal sensitisation or lung toxicity.

### 4.8. Translation and Scalability: Industrial Needleless Electrospinning and Techno-Economic Considerations

Finally, the practical feasibility of the developed solution is highlighted by the use of industrial needleless electrospinning technology, which ensures scalability. Whereas traditional needle-based electrospinning is limited in throughput, needleless electrospinning systems can mass-produce nanofiber membranes for filtration applications [72]. This means the antimicrobial nanofiber filter could be produced in large sheets and cut to fit respirators or air purifier cartridges without significant changes to current production lines. Moreover, from a techno-economic perspective, pristine PA6 nanofiber membranes can be produced at relatively low material cost. The raw polymer cost of PA6 nanofibers deposited at low basis weights (≈0.3 gsm) is on the order of 0.1–0.2 € m^−2^, confirming their suitability for large-area filtration applications.

In contrast, hyperbranched polymers represent high-value specialty materials with a wide market price range, typically varying between 1000 and 10,000 € kg^−1^, depending on molecular architecture, functional groups, and purity. Consequently, incorporation of a small mass fraction of HBP into PA6 nanofiber formulations leads to a slight increase in production cost, potentially doubling (0.4–0.5 € m^−2^) the overall material cost of the functional nanofiber layer. This economic consideration underscores the importance of minimizing HBP loading while maximizing its functional contribution, particularly in scalable, cost-sensitive filtration and protective textile applications.

## 5. Conclusions

This work presents a novel antimicrobial nanofiber filtration system that unites mechanical capture efficiency, antibacterial functionality, and safe-by-design principles in a fully polymer-based, metal-free material. By integrating cationic hyperbranched dendritic polymers into recycled polyamide nanofibers, we developed a self-sterilising filter capable of removing FFP3-grade particles (>99%) while maintaining a low-pressure drop and excellent breathability. The filters demonstrated strong contact-active antibacterial activity against both *Staphylococcus aureus* and *Escherichia coli*, achieved without diffusion or leaching of the active agent. Comprehensive cytocompatibility testing across multiple cell lines and reconstructed human tissue models confirmed the material’s safety for dermal and respiratory contact. The membrane’s nanofibrous structure effectively captures virus-sized aerosols through diffusion and interception, ensuring that viral particles remain immobilised and non-proliferative. The findings validate that hyperbranched and dendritic polymers can serve as sustainable antimicrobial agents capable of replacing traditional metal- or antibiotic-based biocides. Combined with its compatibility with industrial needleless electrospinning, the developed system offers a scalable, reusable, and environmentally responsible solution for respiratory protection and indoor air filtration. Such materials represent a promising pathway toward next-generation, safe, durable, and sustainable filtration technologies that contribute to both public health resilience and circular economy objectives. As future work, the durability of the antibacterial functionality will be evaluated under simulated real-world conditions, including prolonged airflow exposure, humid and wetting–drying environments, and repeated mechanical flexing, to assess the long-term stability and functional retention of the PA6/HBP nanofiber membranes.

## Figures and Tables

**Table 1:** Classification of the biocide effects.

Inhibition (mm)	Growth	Description	Evaluation
>1	No	Inhibition zone above 1 mm, no growth	Satisfactory effect
1–0	No	Inhibition zone up to 1 mm, no growth	
0	No	No inhibition zone, no growth	
0	Light	No inhibition zone, limited growth (few colonies)	Significant effect
0	Moderate	No inhibition zone, reduced growth	No significant effect
0	High	No difference, with the control	

**Table 2:** Characterisation data for hyperbranched polymers and linear-dendritic HBPs (LD-HBPs) used in this study.

Sample ID	Polymeric Architecture	Pseudo-Generation	End Groups	No. of End Groups (Theo.)	No. of End Groups (NMR)	M_n_^theo^ [g mol^−1^]	M_n_^NMR^ [g mol^−1^]
PFH-32-NH_3_^+^	HBP	3	Amine	32	31.4	9540	9460
PFLDHB-G4-PEG10k-NH_3_^+^	LD-HBP	4	Amine	32	28.5	19,400	18,600
PFLDHB-G5-PEG10k-NH_3_^+^	LD-HBP	5	Amine	64	61.7	29,100	28,100
PFLDHB-G5-PEG10k-OH	LD-HBP	5	Hydroxyl	64	61.5	17,200	16,900

**Table 3:** MIC and MBC of the different HBPs determined on *S. aureus* and *E. coli*.

	Concentration (%)			
	*S. aureus*		*E. coli*	
Sample ID	MIC	MBC	MIC	MBC
PFH-32-NH_3_^+^	>0.8	0.8	0.08	0.008
PFLDHB-G4-PEG10k-NH_3_^+^	>0.8	0.8	0.4	0.08
PFLDHB-G5-PEG10k-NH_3_^+^	0.8	0.4	0.8	0.4
PFLDHB-G5-PEG10k-OH	>0.8	>0.8	>0.8	>0.8

**Table 4:** Solution composition and deposition properties.

Sample Code	PA6 Concentration	Type of HBP and Concentration	Winding Speed
PA6-Control	12.5 *w*/*v*	-	1 mm/s
MB47.1	12.5 *w*/*v*	3 wt.% PFLDHB-G5-PEG10k-NH_3_^+^	1 mm/s
MB47.2	12.5 *w*/*v*	6 wt.% PFLDHB-G5-PEG10k-NH_3_^+^	1 mm/s
MB47.3	12.5 *w*/*v*	3 wt.% PFLDHB-G4-PEG10k-NH_3_^+^	1 mm/s
MB47.4	12.5 *w*/*v*	6 wt.% PFLDHB-G4-PEG10k-NH_3_^+^	1 mm/s

**Table 5:** Antimicrobial properties of nanofiber samples.

Sample ID	Nanofiber Composition	Initial Bacterial Concentration (CFU/mL)	Final Bacterial Concentration (CFU/mL)	Exposure Time (h)	Reduction (%)
MB47.2	PA6 + 6 wt.% PFLDHB-G5-PEG10k-NH_3_^+^	1.16 × 10^8^	3.0 × 10^7^	2	74.1%
MB47.4	PA6 + 6 wt.% PFLDHB-G4-PEG10k-NH_3_^+^	1.16 × 10^8^	5.08 × 10^7^	2	56.2%

**Table 6:** Gene expression profiles of pro-inflammatory genes (*IL-6*, *TNF-α*, *PTGS2*), oxidative stress genes (*SOD1*, *CAT*, *OGG1*), and apoptotic genes (*BCL2*, *BAX*, *TP53*) of EpiDerm FT™ and MucilAir™ tissue models. Value in bold formation indicates a significant decrease compared to a control (EpiDerm FT™ with culture medium, MucilAir™ tested without scaffold), and values in italic formation indicate a considerable increase compared to a control.

		EpiDerm FT™				MucilAir™			
		MB47.2		MB47.4		MB47.2		MB47.4	
		FC	*p*-Value	FC	*p*-Value	FC	*p*-Value	FC	*p*-Value
***IL-6***	**24 h**	0.644	0.085	0.884	0.201	**0.539**	0.022	**0.699**	0.021
	**48 h**	1.266	0.275	1.339	0.206				
***TNFalfa***	**24 h**	0.874	0.271	1.141	0.647	0.632	0.053	1.047	0.630
	**48 h**	0.992	0.707	0.865	0.172				
***PTGS2***	**24 h**	0.951	0.205	1.100	0.290	**0.670**	0.007	1.165	0.136
	**48 h**	1.342	0.157	1.541	0.054				
***SOD1***	**24 h**	0.851	0.289	0.964	0.545	*1.355*	0.001	1.119	0.177
	**48 h**	1.145	0.619	0.990	0.694				
***CAT***	**24 h**	1.089	0.283	1.218	0.112	*1.198*	0.026	1.022	0.881
	**48 h**	1.238	0.077	1.196	0.137				
***OGG1***	**24 h**	**0.754**	0.041	**0.772**	0.042	*1.206*	0.004	1.050	0.517
	**48 h**	0.996	0.759	1.040	0.862				
***BCL2***	**24 h**	0.558	0.052	0.792	0.148	1.120	0.400	0.837	0.175
	**48 h**	1.284	0.250	0.995	0.724				
***BAX***	**24 h**	**0.637**	0.005	**0.768**	0.028	1.068	0.378	1.203	0.101
	**48 h**	1.011	0.975	1.003	0.903				
***tp53***	**24 h**	0.951	0.683	1.010	0.964	0.941	0.500	1.030	0.983
	**48 h**	1.295	0.129	1.085	0.786				

**Table 7:** Filtration Test TSI (EN 149:2001 at 95 L/min for 0.3 micrometre particle size).

Sample Code	Composition	Measurement 1		Measurement 2		Measurement 3	
		Pressure Drop (mmH_2_O)	Filtration Efficiency (&)	Pressure Drop (mmH_2_O)	Filtration Efficiency (&)	Pressure Drop (mmH_2_O)	Filtration Efficiency (&)
Control	PA6	37.69	99.98	34.40	99.95	36.42	99.97
MB47.1	PA6 + 3 wt.% PFLDHB PEG10k-G5-NH_3_	52.71	99.995	56.71	99.998	51.22	99.998
MB47.2	PA6 + 6 wt.% PFLDHB PEG10k-G5-NH_3_	36.32	99.89	49.53	99.98	47.97	99.95
MB47.3	PA6 + 3 wt.% PFLDHB PEG10k-G4-NH_3_	37.65	99.79	41.26	99.90	38.64	99.81
MB47.4	PA6 + 6 wt.% PFLDHB PEG10k-G4-NH_3_	48.58	99.92	48.08	99.91	37.65	99.89

**Table 8:** Comparison of commercial surgical or procedure face masks.

Product/Media	Manufacturer	Filtration Efficiency	Pressure Drop	Antibacterial/Antiviral
1820S Splash-Resistant Surgical Mask [79]	3M	99.64 @ 0.3 µm	23.92 Pa/cm^2^	No intrinsic antibacterial agent
FLUIDSHIELD Level 3 [80]	HALYARD	BFE 99.8%; PFE 98.9% @ 0.1 µm	29.42 Pa/cm^2^	No antibacterial claim
DC365 Surgical N95 Respirator [81]	Honeywell	95% @ 0.3 µm	Inhalation < 343 Pa Exhalation < 245 Pa	No antibacterial property
X-plore 1720 (FFP2) family [82]	Dräger	NaCl: 94%; Paraffin oil: 94%	Inhalation: @ 30 L/min 70 Pa; @ 95 L/min 240 Pa. Exhalation: @ 160 L/min 300 Pa	No antibacterial property
ASTM Level 3 procedure mask [83]	Medline	BFE ≥ 99%; PFE > 99%	58.84 Pa/cm^2^	No antibacterial property

## Data Availability

Data are contained within the article.

## Supplementary Materials

The following supporting information can be downloaded at: https://www.mdpi.com/article/10.3390/polym18030374/s1, Figure S1: Oxidative stress was measured as the produced H_2_O_2_ in the culture of (a) HaCaT keratinocytes, (b) Melan-a melanocytes, and (c) BEAS-2B bronchial epithelial cells cultivated in extracts from MB47.2 and MB47.4 materials for 1.5 h. Culture medium served as a negative control. Culture medium containing 750 µM H_2_O_2_ served as a positive control. The statistical difference between the groups is marked as a line. **** *p* < 0.0001.
