# Supplementary material for: Hyperbranched Polymer Dendrimers Embedded in Electrospun Nanofibers for Safe and Sustainable Antibacterial Filtration Materials
Source: Polymers (Basel). 2026 Jan 30;18(3):374. doi: 10.3390/polym18030374 (PMC12899854; doi:10.3390/polym18030374)
Supplement: Supplementary file 1 [file polymers-18-00374-s001.zip › polymers-4044426-supplementary.pdf]

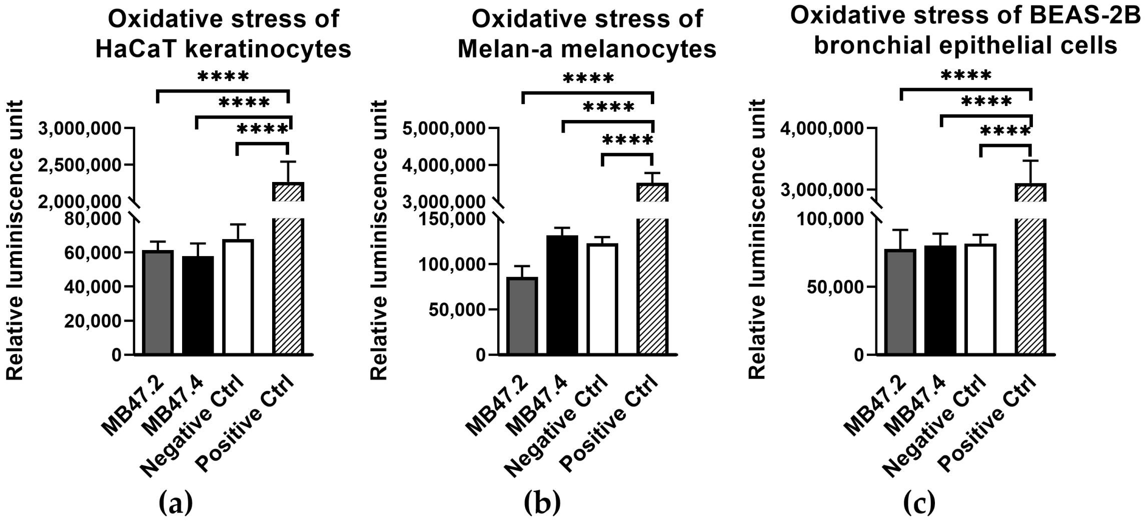

Figure S1: Oxidative stress was measured as the produced H2O2 in the culture of (**a**) HaCaT keratinocytes, (**b**) Melan-a melanocytes, and (**c**) BEAS-2B bronchial epithelial cells cultivated in extracts from MB47.2 and MB47.4 materials for 1.5 h. Culture medium served as a negative control. Culture medium containing 750 μM H2O2 served as a positive control. The statistical difference between the groups is marked as a line. **** $p < 0.0001$.
